# Preparative Separation of Diterpene Lactones and Flavones from *Andrographis paniculate* Using Off-Line Two-Dimensional High-Speed Counter-Current Chromatography

**DOI:** 10.3390/molecules24030620

**Published:** 2019-02-11

**Authors:** Xiaowei Sun, Huijiao Yan, Yujie Zhang, Xiao Wang, Dawei Qin, Jinqian Yu

**Affiliations:** 1 Shandong Key Laboratory of TCM Quality Control Technology, Shandong Analysis and Test Center, Qilu University of Technology (Shandong Academy of Sciences), Jinan 250014, China; sxw199301@163.com (X.S.); yanhuijiao01@163.com (H.Y.); wangx@sdas.org (X.W.); 2 School of Chemistry and Pharmaceutical Engineering, Qilu University of Technology (Shandong Academy of Sciences), Jinan 250353, China; 3 School of Life Sciences, Linyi University, Linyi 276000, China; zhangyujie@lyu.edu.cn

**Keywords:** *Andrographis paniculata* (Burm.f.) Nees, off-line two-dimensional high-speed counter-current chromatography, diterpene lactones, flavones

## Abstract

Seven diterpene lactones, andrographolide (**1**), isoandrographolide (**2**), neo-andrographolide (**3**), 14-deoxy-11,12-didehydroandrographolide (**4**), 14-deoxyandrographiside (**5**), 14-deoxy-11,12-didehydroandrographiside (**6**), 3,14-dideoxyandrographolide (**10**), and three flavones, andrographidine C (**7**), andrographidine A (**8**), 5-hydroxy-7,8-dimethoxyflavanone (**9**) have been successfully and efficiently isolated from *A. paniculata* using an off-line two dimensional (2D) high-speed counter-current chromatography (HSCCC) method for the first time. For the first dimension HSCCC separation, petroleum ether-ethyl acetate-methanol-water 3:7:5:5 (*v*/*v*) was employed to isolate 14.4 mg of compound **1**, 3.1 mg of compound **2**, 7.8 mg of compound **3**, and 18.0 mg of compound **4** from 200 mg of the *A. paniculata* extract. For the second dimension HSCCC separation, petroleum ether-ethyl acetate-methanol-water 2:8:1:9 (*v*/*v*) and 5:5:6:4 (*v*/*v*) were employed to isolate the collected fractions ranged from 55 to 79 min and the flow out fraction, respectively, which led to 5.1 mg of compound **5**, 4.4 mg of compound **6**, 2.4 mg of compound **7**, 3.3 mg of compound **8**, 4.0 mg of compound **9**, 7.0 mg of compound **10**. The structures of these diterpene lactones and flavones were elucidated by extensive spectroscopic methods.

## 1. Introduction

Traditional Chinese medicine has received more and more attention due to their structurally complex compounds and multiple pharmacological activities. Accordingly, various chromatographic isolated methods have been developed to purify compounds from the natural medicine, such as conventional open column chromatography, preparative column chromatography, and so on. High-speed counter-current chromatography (HSCCC), a recently appeared liquid–liquid partition chromatography technique, is used more and more widely in the purification of complex compounds from natural products not only because of its no irreversible adsorption of constituents on account of lacking a solid support matrix but also due to its large sample loading size and high separation efficiency. 

The genus *Andrographis*, belonging to the family of *Acanthaceae*, is usually distributed in the tropical and subtropical regions. About 20 species have been identified as this genus, of which five species were found in China. *Andrographis paniculata* (Burm.f.) Nees is one of the five species, and the leaves and stems of which have been recorded in the Chinese Pharmacopoeia to treat fever, swollen sore throat, poisonous snake bite, and so on [1]. Previous phytochemical literatures of *A. paniculata* have focused on the isolation and identification of diterpene lactones, flavones, and steroids [2,3], with the first two kinds being well-known for possessing diverse activities, including anti-microbial, anti-inflammatory, anticoagulation, hepatoprotective, anti-fertility, and immunoregulation [4,5,6,7]. The main active and characteristic diterpene lactones are reported as andrographolide, 14-deoxy-11,12-didehydroandrographolide, and neoandrographolide, among which andrographolide has been accepted as the most active constituent of *A. paniculata* [6]. Nowadays, the quality control of this medicinal plant needs to be appreciated due to different planting environments, picking times, and storage conditions. Isolation and identification of the main components from this plant is of great significance, which can serve as maker compounds to control the quality of *A. paniculata*. However, currently available methods for the separation of these active compounds, such as column chromatography separation, often renders to high consumption of separating packing materials, solvent, and time, and irreversible adsorption of the larger polar compounds [2,3,8].

**Figure 1:**
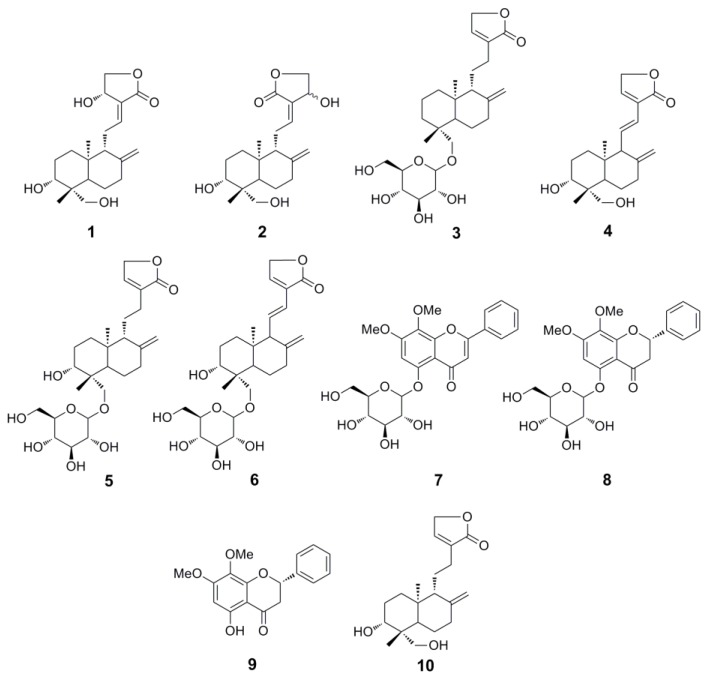
Chemical structures of compounds **1**–**10**.

Consequently, a rapid separation method for the diterpene lactones and flavones needs to be established urgently. High-speed counter-current chromatography (HSCCC) could be promising due to its advantages mentioned above. However, the traditional unidimensional HSCCC separation for complex extracts usually leads to the acquisition of a small number of purified compounds. To obtain more compounds, a newly developed strategy, two-dimensional (2D) HSCCC, has been used, which has been divided into two main classes: on-line and off-line 2D HSCCC, the former usually needs two HSCCC separation apparatus, and the latter is much easier [9]. Herein, an off-line 2D HSCCC method was successfully applied for the separation of seven diterpene lactones, andrographolide (**1**), isoandrographolide (**2**), neo-andrographolide (**3**), 14-deoxy-11,12-didehydroandrographolide (**4**), 14-deoxyandrographiside (**5**), 14-deoxy-11,12-didehydroandrographiside (**6**), 3,14-dideoxyandrographolide (**10**), and three flavones, andrographidine C (**7**), andrographidine A (**8**), 5-hydroxy-7,8-dimethoxyflavanone (**9**), from *A. paniculata* (Figure 1). 

## 2. Results

### 2.1. Optimization of HPLC Conditions

**Figure 2:**
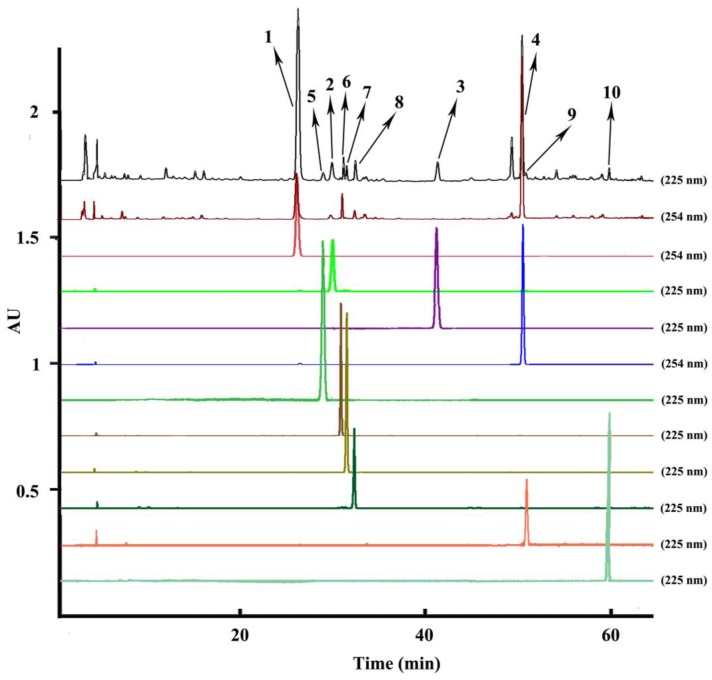
HPLC chromatograms of the extraction sample and peak fractions from *Andrographis paniculata*. HPLC conditions: column: Eclipse Plus C8 (250 mm × 4.6 mm i.d., 5 μm); mobile phase: acetonitrile (A), water (B), 0–5 min, 20–20% A; 5–10 min, 20–23% A; 10–25 min, 23–26%A; 26-40 min, 31–31%A; 41–60 min, 31–60%A; 61-70 min, 60–70%A; 71–80 min, 100–100%A; column temperature: 25 °C; flow rate: 1.0 mL min^−1^; UV detection wavelength: both 225 nm and 254 nm for the extraction sample, 225 nm for peaks 2, 3, 5–10, and 254 nm for the peaks 1 and 4; injection volume: 10 μL; peak 1: andrographolide, peak 2: isoandrographolide, peak 3: neo-andrographolide, peak 4: 14-deoxy-11,12-didehydroandrographolide, peak 5: 14-deoxyandrographiside, peak 6: 14-deoxy-11,12-didehydroandrographiside, peak 7: andrographidine C, peak 8: andrographidine A, peak 9: 5-hydroxy-7,8-dimethoxyflavanone, and peak 10: 3,14-dideoxyandrographolide.

Optimization of the HPLC conditions was carried out with different mobile phases including acetonitrile/water, acetonitrile/water acidified with 0.1% acetic acid *v*/*v*, acetonitrile/water acidified with 0.1% phosphoric acid *v*/*v*, acetonitrile/water acidified with 0.1% formic acid *v*/*v*, different gradient elution modes, and different columns. When a normal C18 analytical column was used with the mobile phase of acetonitrile/water, the fronting peaks appeared in the HPLC chromatography of the *A. paniculata* crude extract. To overcome this problem, 0.1% acetic acid, 0.1% phosphoric acid, and 0.1% formic acid were added into the water to improve the peak shapes. However, the fronting peaks in the chromatography hardly changed. Consequently, a C8 analytical column was used to improve the fronting shapes of the peaks, which could reduce the adsorption of the sample to eliminate the fronting peaks. Finally, the analytical column was determined as Eclipse Plus C8 and the optimized HPLC mobile phase was composed of acetonitrile/water with a suitable gradient elution mode set at 0–5 min, 20–20%A; 5–10 min, 20–23%A; 10–25 min, 23–26%A; 26–40 min, 31–31%A; 41–60 min, 31–60%A; 61–70 min, 60–70%A; 71–80 min, 100–100%A, at a flow rate of 1.0 mL/min. The flow rate, injection volume and temperature were set as 1.0 mL/min, 10 μL and 25 °C. Spectra were detected from 190 to 400 nm with the chromatogram analyzing at 225 nm and 254 nm (the upper two chromatograms in Figure 2). Under the optimized HPLC conditions, the ten separated compounds (the bottom ten chromatograms in Figure 2) reached baseline separation and were identified as the relative compounds **1**–**10** in the upper two HPLC chromatograms of the extraction sample according to the same retention time.

### 2.2. Optimization of the High-Speed Counter-Current Chromatography Conditions

**Figure 3:**
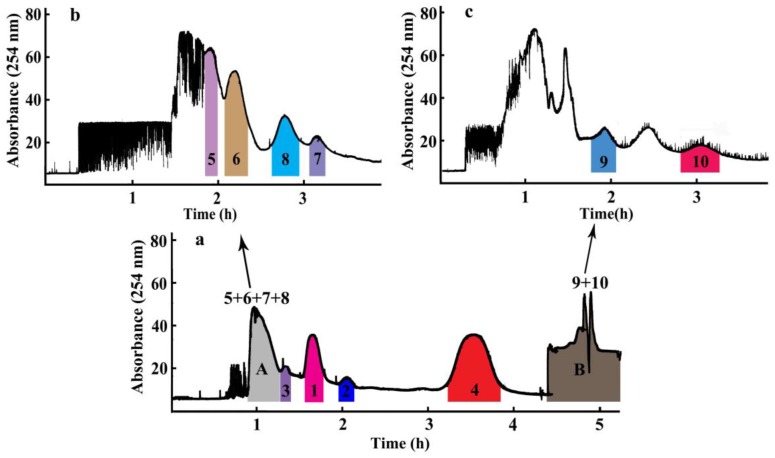
The off-line two-dimensional (2D) high-speed counter-current chromatography (HSCCC) chromatogram for the separation of *Andrographis paniculata* extract. (**a**) Chromatogram of the first dimension HSCCC separation: solvent system, petroleum ether-ethyl acetate-methanol-water (3:7:5:5, *v*/*v*). (**b**) Chromatogram of the second dimension HSCCC separation of part A: solvent system, petroleum ether-ethyl acetate-methanol-water (2:8:1:9, *v*/*v*). (**c**) Chromatogram of the second dimension HSCCC separation of part B: solvent system, petroleum ether-ethyl acetate-methanol-water (5:5:6:4, *v*/*v*). Other experimental conditions: the upper organic phase as the stationary phase and the lower aqueous phase as the mobile phase; revolution speed: 800 rpm; flow rate: 2.0 mL min^−1^; sample size: 200 mg; UV detection wavelength: 254 nm; retention of stationary phase: 60.0% (**a**), 44.4% (**b**), and 63.3% (**c**).

Ten compounds, including seven diterpene lactones, andrographolide (**1**), isoandrographolide (**2**), neo-andrographolide (**3**), 14-deoxy-11,12-didehydroandrographolide (**4**), 14-deoxyandrographiside (**5**), 14-deoxy-11,12-didehydroandrographiside (**6**), 3,14-dideoxyandrographolide (**10**), and three flavones, andrographidine C (**7**), andrographidine A (**8**), 5-hydroxy-7,8-dimethoxyflavanone (**9**), from *A. paniculata* were isolated over periods that ranged broadly from 25 min to 60 min with a mobile phase between 20% acetonitrile/water and 60% acetonitrile/water in the HPLC analysis. Owing to the different polarities of these target compounds, selection of an excepted two-phase solvent system was most important of all during a HSCCC separation, which can be achieved when the tested partition coefficient (*K*_D_) values for the target compounds range from 0.5 to 2.0 [10,11]. According to the previous reports and separation experiences for compounds with large differences of polarities, the solvent system composed of petroleum ether-ethyl acetate-ethanol/methanol-water was employed as the experimental one [12,13]. A series of *K*_D_ values testing experiments were performed against three solvent systems with solvent ratios of petroleum ether-ethyl acetate-ethanol-water as 2:8:3:7, 2:8:5:5, and petroleum ether-ethyl acetate-methanol-water as 3:7:5:5 with *K*_D_ values for the ten compounds listed in Table 1. Furthermore, results demonstrated that when ethanol was changed to methanol and volume ratios of these four solvents were 3:7:5:5, the suitable *K*_D_ values for compounds **1**–**4** were obtained. According to the *K*_D_ value rules of selecting the appropriate solvent system, when the solvent system of petroleum ether-ethyl acetate-methanol-water as (3:7:5:5, *v*/*v*) was tested, compounds **1**–**4** were successfully separated as four peaks by HSCCC with compounds **5**–**8** co-eluted in the first peak due to their nearly the same *K*_D_ values and compounds **9** and **10** flowed out together at the end of the separation due to their much larger *K*_D_ values, as shown in Figure 3. Owing to the poor peak resolutions of these other six compounds, an off-line 2D HSCCC method was applied to increase their peak capacities and clearly separate them. First, the co-eluted compounds **5**–**8** and **9**–**10** in Figure 3 were collected as part A and part B, respectively, which were concentrated to dryness for subsequent 2D HSCCC separation. Second, the enriched part for compounds **5**–**8** was subjected to three more hydrophilic solvent systems with different volume ratios of petroleum ether-ethyl acetate-methanol-water as 3:7:4:6, 3:7:3:7, and 2:8:1:9. The *K*_D_ values for compounds **5**–**8** in Table 1 indicated the solvent system of petroleum ether-ethyl acetate-methanol-water (2:8:1:9, *v*/*v*) was suitable for the HSCCC separation for these four compounds. Finally, the enriched part for compounds **9** and **10** was subjected to one less hydrophilic solvent system of petroleum ether-ethyl acetate-methanol-water (5:5:6:4, *v*/*v*), with suitable *K*_D_ values for compounds **9** and **10** assigned. Therefore, three solvent systems of petroleum ether-ethyl acetate-methanol-water (3:7:5:5, 2:8:1:9, 5:5:6:4, *v*/*v*) were ultimately applied to separate compounds **1**–**4**, **5**–**8**, and **9**–**10**, respectively, using an off-line 2D HSCCC separation strategy. 

Moreover, factors including the flow rate of the solvent (1.0, 2.0, and 2.5 mL/min), and the revolution speed of the separation column (700, 800, and 900 rpm) were also investigated. Clearly, a decreased flow rate and increased revolution speed could both result in a higher percent of retention of the stationary phase; however, with the former lengthening the separation time, and the latter broadening the sample band. In view of the above-mentioned influence, the flow rate of 2.0 mL/min, and the revolution speed of 800 rpm were employed to run the off-line 2D HSCCC separation.

### 2.3. Purification of Diterpene Lactones and Flavones by 2D-HSCCC

Two-hundred milligrams of *A. paniculata* crude extract was successfully separated by an off-line 2D HSCCC separation using the solvent system of petroleum ether-ethyl acetate-methanol-water with three different ratios of 3:7:5:5, 2:8:1:9, and 5:5:6:4. As shown in Figure 3a, four compounds (**1**–**4**) were successfully separated in the 1D HSCCC by the solvent system of petroleum ether-ethyl acetate-methanol-water (3:7:5:5, *v*/*v*). However, the relatively close *K*_D_-values of compounds **5** (0.12), **6** (0.15), **7** (0.23), and **8** (0.22) rendered these four compounds co-eluted as one peak in part A, and the relatively smaller polarities of compounds **9** and **10** rendered these two compounds mainly distributed in the upper phase, which were eluted together in part B at the end of the separation. Consequently, an off-line 2D HSCCC method was applied to increase their peak capacities and clearly separate them. First, part A (55 min to 79 min) and part B (the flow out fraction) in the 1D HSCCC were concentrated to dryness for the subsequent 2D HSCCC separation. Second, the enriched part A for compounds **5**–**8** was subjected to one more hydrophilic solvent system of petroleum ether-ethyl acetate-methanol-water (2:8:1:9, *v*/*v*), and four peaks corresponding to compounds **5**–**8** appeared in the 2D HSCCC chromatogram of Figure 3b. Subsequently, the enriched part B for compounds **9** and **10** was subjected to one less hydrophilic solvent system of petroleum ether-ethyl acetate-methanol-water (5:5:6:4, *v*/*v*), and two peaks corresponding to compounds **9** and **10** appeared in the 2D HSCCC chromatogram of Figure 3c. Finally, after the off-line 2D HSCCC separation run, 14.4 mg of andrographolide (**1**), 3.1 mg of isoandrographolide (**2**), 7.8 mg of neo-andrographolide (**3**), 18.0 mg of 14-deoxy-11,12-didehydroandrographolide (**4**), 5.1 mg of 14-deoxyandrographiside (**5**), 4.4 mg of 14-deoxy-11,12-didehydroandrographiside (**6**), 2.4 mg of andrographidine C (**7**), 3.3 mg of andrographidine A (**8**), 4.0 mg of 5-hydroxy-7,8-dimethoxyflavanone (**9**), and 7.0 mg of 3,14-dideoxyandrographolide (**10**), were obtained from 200 mg of the *A. paniculata* crude extract, with purities all over 90.0% determined using HPLC. 

### 2.4. Structure Identification of the Isolated Compounds

The structure elucidations of compounds **1**-**10** were finally achieved by comparing the HRESI-MS and NMR spectroscopic data with those in literatures [2,8,14,15,16,17] (NMR and MS spectroscopic data for compounds **1**–**10**, supplementary data). Finally, compounds **1**–**10** were identified as andrographolide (**1**), isoandrographolide (**2**), neo-andrographolide (**3**), 14-deoxy-11,12-didehydroandrographolide (**4**), 14-deoxyandrographiside (**5**), 14-deoxy-11,12-didehydroandrographiside (**6**), andrographidine C (**7**), andrographidine A (**8**), 5-hydroxy-7,8-dimethoxyflavanone (**9**), and 3,14-dideoxyandrographolide (**10**), respectively.

*Andrographolide* (**1**): HRESI-MS (*m/z*): 351.1820 [M + H]^+^. ^1^H-NMR (pyridine-*d*_5_) *δ*: 7.16 (1H, td, *J* = 6.1, 1.9 Hz, H-12), 5.40 (1H, brs, H-14), 4.50(1H, dd, *J* = 10.0, 2.8 Hz, H-15a), 4.60 (1H, dd, *J* = 10.0, 7.0 Hz, H-15b), 4.84 (1H, brs, H-17a), 4.86(1H, brs, H-17b), 1.49 (3H, s, Me-18), 3.60 (1H, overlapped, H-19a), 4.43 (1H, d, *J* = 10.8 Hz, H-19b), 0.65 (3H, s, Me-20). ^13^C-NMR (pyridine-*d*_5_) *δ*: 37.1 (C-1), 28.8 (C-2), 79.7 (C-3), 43.1 (C-4), 55.1 (C-5), 24.2 (C-6),38.0 (C-7), 147.8 (C-8), 56.2 (C-9), 39.0 (C-10), 24.8 (C-11), 146.8(C-12), 130.0 (C-13), 65.8 (C-14), 75.2 (C-15), 170.5 (C-16), 108.6 (C-17), 23.5 (C-18), 64.0 (C-19), 15.0 (C-20).

*Isoandrographolide* (**2**): HRESI-MS (*m/z*): 351.2445 [M + H]^+^. ^1^H-NMR (pyridine-*d*_5_) *δ*: 6.70 (1H, brt, *J* = 7.0 Hz, H-12), 5.05 (1H, brs, H-14), 4.38 (1H, dd, *J* = 10.0, 3.2 Hz, H-15a), 4.51 (1H, dd, *J* = 10.0, 7.0 Hz, H-15b), 4.71 (1H, brs, H-17a), 4.87 (1H, brs, H-17b), 1.49 (3H, s, Me-18), 3.60 (1H, m, H-19a), 4.44 (1H, d, *J* = 10.8 Hz, H-19b), 0.71 (3H, s, Me-20). ^13^C-NMR (pyridine-*d*_5_) *δ*: 37.1 (C-1), 28.8 (C-2), 79.7 (C-3), 43.0 (C-4), 55.1 (C-5), 24.3 (C-6), 38.1 (C-7), 148.1 (C-8), 56.5 (C-9), 39.2 (C-10), 23.5 (C-11), 148.0 (C-12), 129.6 (C-13), 68.9 (C-14), 74.1 (C-15), 169.7 (C-16), 107.9 (C-17), 23.5 (C-18), 64.0 (C-19), 15.1 (C-20).

*Neo-andrographolide* (**3**): HRESI-MS (*m/z*): 503.2412 [M+Na]^+^. ^1^H-NMR (pyridine-d_5_) *δ*: 7.15 (1H, brs, H-14), 4.70 (1H, s, H-17a), 4.89 (1H, s, H-17b), 1.19 (3H, s, H-18), 3.49 (1H, d, *J* = 9.6 Hz, H-19a), 4.33 (1H, d, *J* = 9.6 Hz, H-19b), 0.65 (3H, s, Me-20), 4.83 (1H, d, *J* = 8.0 Hz, Glu-1′). ^13^C-NMR (pyridine-*d*_5_) *δ*: 38.9 (C-1), 19.2 (C-2), 36.2 (C-3), 39.6 (C-4), 56.0 (C-5), 24.5 (C-6), 38.6 (C-7), 148.0 (C-8), 56.5 (C-9), 38.4 (C-10), 21.9 (C-11), 24.8 (C-12), 134.0 (C-13), 145.1 (C-14), 70.38 (C-15), 174.4 (C-16), 106.7 (C-17), 28.0 (C-18), 72.4 (C-19), 15.2 (C-20), 105.3 (Glu-1′), 75.2 (Glu-2′), 78.5 (Glu-3′), 71.6 (Glu-4′), 78.2 (Glu-5′), 62.7 (Glu-6′).

*14-deoxy-11,12-didehydroandrographolide* (**4**): HRESI-MS (*m/z*): 355.1765 [M + Na]^+^. ^1^H-NMR (pyridine-*d*_5_) *δ*: 7.16 (1H, dd, *J* = 10.0, 15.6 Hz, H-11), 6.26 (1H, dd, *J* = 16.0 Hz, H-12), 7.31 (1H, brs, H-14), 4.78 (2H, brs, H-15), 4.74 (1H, d, *J* = 1.2 Hz, H-17a), 4.85 (1H, d, *J* = 1.2 Hz, H-17b), 1.51 (3H, s, Me-18), 3.65 (1H, d, *J* = 8.0 Hz, H-19a), 4.48 (1H, d, *J* = 10.8 Hz, H-19b), 0.88 (3H, s, Me-20). ^13^C-NMR (pyridine-*d*_5_) *δ*: 38.1 (C-1), 28.0 (C-2), 79.2 (C-3), 42.4 (C-4), 53.9 (C-5), 22.7 (C-6), 36.1 (C-7), 148.1 (C-8), 60.9 (C-9), 37.8 (C-10), 121.0 (C-11), 134.8 (C-12), 128.0 (C-13), 144.1 (C-14), 69.4 (C-15), 171.9 (C-16), 107.8 (C-17), 22.7 (C-18), 63.3 (C-19), 14.1 (C-20).

*14-deoxyandrographiside* (**5**): HRESI-MS (*m/z*): 519.2346 [M + Na]^+^. ^1^H-NMR (pyridine-*d*_5_) *δ*: 7.16 (1H, brs, H-14), 4.72 (3H, overlapped, H_2_-15, H-17a), 4.90 (1H, brs, H-17b), 1.46 (3H, s, Me-18), 3.88 (1H, d, *J* = 10.0 Hz, H-19a), 4.67 (1H, d, *J* = 10.0 Hz, H-19b), 0.83 (3H, s, Me-20), 4.85 (1H, *J* = 7.6 Hz, Glu-1′), 4.38 (1H, dd, *J* = 11.1, 5.1 Hz, Glu-6′a), 4.52 (1H, brd, *J* = 11.1 Hz, Glu-6′b). ^13^C-NMR (pyridine-*d*_5_) *δ*: 37.6 (C-1), 29.0 (C-2), 78.8 (C-3), 43.1 (C-4), 55.5 (C-5), 25.2 (C-6), 38.6 (C-7), 148.0 (C-8), 56.4 (C-9), 39.5 (C-10), 22.1 (C-11), 24.8 (C-12), 134.0 (C-13), 145.2 (C-14), 70.4 (C-15), 174.4 (C-16), 106.8 (C-17), 24.3 (C-18), 72.0 (C-19), 14.7 (C-20), 105.4 (C-1′), 74.7 (C-2′), 78.6 (C-3′), 71.4 (C-4′), 78.4 (C-5′), 62.5 (C-6′).

*14-deoxy-11,12-didehydroandrographiside* (**6**): HRESI-MS (*m/z*): 517.2218 [M + Na]^+^. ^1^H-NMR (pyridine-*d*_5_) *δ*: 7.16 (1H, overlapped, H-11), 6.25 (1H, d, *J* = 16.0 Hz, H-12), 7.28 (1H, brs, H-14), 4.76 (1H, s, H-17a), 4.83 (1H, s, H-17b), 1.48 (3H, s, H-18), 3.92 (1H, d, *J* = 10.4 Hz, H-19a), 4.67 (1H, d, *J* = 10.4 Hz, H-19b), 1.06 (3H, s, Me-20), 4.84 (1H, d, *J* = 7.6 Hz, Glu-1′). ^13^C-NMR (pyridine-*d*_5_) *δ*: 38.8 (C-1), 28.7 (C-2), 78.8 (C-3), 42.9 (C-4), 54.6 (C-5), 24.0 (C-6), 36.8 (C-7), 149.2 (C-8), 61.6 (C-9), 38.9 (C-10), 135.5 (C-11), 120.4 (C-12), 128.5 (C-13), 143.4 (C-14), 69.8 (C-15), 172.4 (C-16), 108.1 (C-17), 24.2 (C-18), 71.9 (C-19), 15.1 (C-20), 105.1 (Glu-1′), 76.5 (Glu-2′), 78.4 (Glu-3′), 71.2 (Glu-4′), 78.2 (Glu-5′), 62.3 (Glu-6′).

*Andrographidine C* (**7**): HRESI-MS (*m/z*): 483.1261 [M + Na]^+^. ^1^H-NMR (pyridine-*d*_5_) *δ*: 6.92 (1H, s, H-3), 7.18 (1H, s, H-6), 7.52–7.75 (3H, m, H-3′,4′,5′), 8.06 (2H, m, H-2′,6′), 3.95 (3H, s, 7-OCH_3_), 3.90 (3H, s, 8-OCH_3_), 4.75 (1H, d, *J* = 7.5 Hz, H-1″), 3.33 (1H, m, H-2″), 3.07 (1H, m, H-3″), 3.16 (1H, m, H-4″), 3.23 (1H, m, H-5″), 3.59 (1H, dd, *J* = 3.6, 12.0Hz, H-6″a), 3.33 (1H, m, H-6″b). ^13^C-NMR (pyridine-*d*_5_) *δ*: 161.5 (C-2), 108.4 (C-3), 178.1 (C-4), 110.6 (C-4a), 154.6 (C-5), 101.9 (C-6), 133.4 (C-8), 151.2 (C-8a), 133.4 (C-1′), 126.4 (C-2′), 129.2 (C-3′), 131.7 (C-4′), 129.2 (C-5′), 126.4 (C-6′), 106.6 (Glu-1′), 75.1 (Glu-2′), 79.5 (Glu-3′), 71.5 (Glu-4′), 77.7 (Glu-5′), 62.6 (Glu-6′), 56.1 (7-OCH_3_), 61.1(8-OCH_3_).

*Andrographidine A* (**8**): HRESI-MS (*m/z*): 485.1224 [M + Na]^+^. ^1^H-NMR (pyridine-*d*_5_) *δ*: 5.47 (1H, dd, *J* = 2.8, 12.8 Hz, H-2), 3.04 (1H, dd, *J* = 13.2, 15.2 Hz, H-3a), 2.92 (1H, dd, *J* = 3.2, 15.2 Hz, H-3b), 7.27 (1H, s, H-6), 7.59 (2H, d, *J* = 7.2 Hz, H-2′,6′), 7.40 (2H, t, *J* = 7.2 Hz, H-3′, 5′), 7.33 (1H, m, H-4′), 5.34 (1H, d, *J* = 7.2 Hz, H-Glu-1′), 3.85 (3H, s, 7-OCH_3_), 3.80 (3H, s, 8-OCH_3_). ^13^C-NMR (pyridine-*d*_5_) *δ*: 78.1 (C-2), 44.5 (C-3), 189.2 (C-4), 106.7 (C-4a), 158.2 (C-5), 95.6 (C-6), 155.5 (C-7), 131.9 (C-8), 155.0 (C-8a), 138.1 (C-1′), 125.2 (C-2′, 6′), 127.7 (C-3′, 5′), 127.5 (C-4′), 104.3 (Glu-1′), 73.7 (Glu-2′), 78.3 (Glu-3′), 70.3 (Glu-4′), 76.5 (Glu-5′), 61.3 (Glu-6′), 54.8 (7-OCH_3_), 58.4 (8-OCH_3_).

*5-hydroxy-7,8-dimethoxyflavanone* (**9**): HRESI-MS (*m/z*): 335.1707 [M-Cl]^-^. ^1^H-NMR (pyridine-*d*_5_) *δ*: 5.47 (1H, dd, *J* = 12.4, 3.2 Hz, H-2), 3.07 (1H, dd, *J* = 17.2, 12.4 Hz, H-3a), 2.90 (1H, dd, *J* = 17.2, 3.2 Hz, H-3b), 6.12 (1H, s, H-6), 7.45 (5H, m, H-2′, H-3′, H-4′, H-5′, H-6′), 3.90 (3H, s, 7-OCH_3_), 3.79 (3H, s, 8-OCH_3_), 12.0 (1H, s, 5-OH). ^13^C-NMR (pyridine-*d*_5_) *δ*: 79.2 (C-2), 45.9 (C-3), 188.0 (C-4), 107.9 (C-4a), 158.9 (C-5), 90.4 (C-6), 158.1 (C-7), 122.7 (C-8), 156.4 (C-8a), 139.7 (C-1′), 126.4 (C-2′, 6′), 128.8 (C-3′, 5′), 128.5 (C-4′), 55.8 (7-OCH_3_), 55.9 (8-OCH_3_).

*3,14-dideoxyandrographolide* (**10**): HRESI-MS (*m/z*): 333.1679 [M-H]^-^. ^1^H-NMR (pyridine-*d*_5_) *δ*: 7.16 (1H, td, *J* = 6.1, 1.9 Hz, H-12), 5.40 (1H, brs, H-14), 4.50 (1H, dd, *J* = 10.0, 2.5 Hz, H-15a), 4.60 (1H, dd, *J* = 10.0, 6.0 Hz, H-15b), 4.84 (1H, brs, H-17a), 4.86 (1H, brs, H-17b), 1.49 (3H, s, Me-18), 3.60 (1H, overlapped, H-19a), 4.43 (1H, d, *J* = 10.5 Hz, H-19b), 0.65 (3H, s, Me-20). ^13^C-NMR (pyridine-*d*_5_) *δ*: 39.2 (C-1), 19.2 (C-2), 35.8 (C-3), 39.0 (C-4), 56.1 (C-5), 24.6 (C-6), 38.7 (C-7), 148.2 (C-8), 56.6 (C-9), 39.7 (C-10), 22.0 (C-11), 24.8 (C-12), 134.0 (C-13), 145.1 (C-14), 70.4 (C-15), 174.4 (C-16), 106.7 (C-17), 27.8 (C-18), 63.6 (C-19), 15.3 (C-20).

## 3. Materials and Methods

### 3.1. Reagents and Materials

*A. paniculata* was purchased from Zhonglu hospital of Shandong University of Traditional Chinese Medicine and authenticated by Prof. Jia Li (Shandong University of Traditional Chinese Medicine). The 95% ethanol, petroleum ether (60–90 °C), ethyl acetate, and methanol used to extract the herb and run HSCCC separation were of analytical grade and purchased from Sinopharm Chemical Reagent Co., Ltd (Shanghai, China). The only solvent of analytical purity was acetonitrile, which was used for HPLC analysis and purchased from Fisher Scientific (Tedia Company, Fairfield, OH, USA). An osmosis Milli-Q system (Millipore, Bedford, MA, USA) was used to prepare the deionized water used for HSCCC and HPLC.

### 3.2. Apparatus

The off-line 2D HSCCC separation were performed with a TBE-300A high-speed counter-current chromatography equipment (Tauto Biotechnique, Shanghai, China), fitted with a multilayer column coil of polytetrafluoroethylene (PTFE) (300 mL of capacity, 1.6 mm in diameter), and a 20 mL manual sample loop. The revolution speed of the column coil was regulated to be 800 rpm. The HSCCC system was equipped with a TBP-5002 constant-flow pump (Tauto Biotechnique, Shanghai, China), an 8823A-UV detector at 254 nm (Beijing Emilion Technology, Beijing, China), a Model 3057 portable recorder (Yokogawa, Sichuan Instrument Factory, Sichuan, China), and a DC-0506 low constant temperature-circulating bath (Tauto Biotechnique, Shanghai, China) to maintain the temperature at 25 °C.

The HPLC analysis of the crude extract and the HSCCC fractions were performed on a Waters e2695 (Waters Corporation, Milford, MA, USA), which consisted of a Waters 2695 solvent delivery unit, a Waters 2998 photodiode array detection (DAD) detector, an autosampler, and a Waters 2695 column oven. Analysis results were collected with an Empower 3 ChemStation unit (Waters Corporation, Milford, MA, USA). An Eclipse Plus C8 (250 mm × 4.6 mm, 5 μm) analytical column (Agilent Technologies Co. Ltd., Palo Alto, CA, USA) was used to perform the HPLC analysis. A Bruker AV-400 spectrometer (Bruker BioSpin, Rheinstetten, Germany) and a Bruker Impact II mass spectrometer (Bruker Daltonic Inc., USA) were employed to perform the NMR analysis and HRESI-MS analysis.

### 3.3. Preparation of Crude Sample

The chopped leaves and stems of *A. paniculata* (200 g) were extracted with 85% aqueous EtOH twice (2 h, 1.5 h) under reflux conditions. After extraction, the extracts were combined and concentrated to remove the solvent with a rotary evaporator. Finally, 30.4 g of 85% ethanol crude extract was obtained and stored at 2–8 °C, which was prepared for the subsequent HSCCC separation.

### 3.4. Preparation of Two-phase Solvent Systems and Sample Solution

For the off-line 2D HSCCC isolation, solvent systems of petroleum ether-ethyl acetate-methanol-water with different volume ratios were prepared. A separatory funnel was used to separate the two-phase solvent system with the upper one as the stationary phase and the lower one as the mobile phase, which can be used until the equilibrium was achieved. The sample solution was prepared by adding 0.2 g of 85% ethanol crude extract into the mixed solvent of the lower phase and upper phase (5 mL for each phase). 

### 3.5. HSCCC Separation Procedure

For the off-line 2D HSCCC experiment, TBE-300A HSCCC equipment was used by the chosen solvent system of petroleum ether-ethyl acetate-methanol-water with different volume ratios. Additionally, the 1D HSCCC and 2D HSCCC separation were both normal HSCCC with the same separation procedures. First, the column coil was entirely filled with the upper phase at 20.0 mL/min, and subsequently rotated at a speed of 800 rpm. Second, the lower phase was pumped into the column at 2.0 mL/min. Third, the sample solvent (each 5 mL for upper and lower phase) containing 200 mg of extract was injected into the apparatus through the sample loop, when the hydrodynamic equilibrium of the solvent system was reached in the coil, along with the lower phase being pumped into the column with a constant flow rate of 2.0 mL/min. HSCCC effluents of the extract were monitored at 254 nm using UV, and collected together every 10 mL. 

Due to the restricted peak capacity of the 1D HSCCC separation, the collected fractions ranged from 55 to 79 min and the flow out fraction were both proceeded with the 2D HSCCC separation with the same procedures presented above. 

At the end, when the 1D and 2D HSCCC separations were over, ethanol was used as the mobile phase to completely elute the residual solvents to calculate the stationary phase retention.

### 3.6. HPLC Analysis and Identification of the Fractions

A Waters e2695 apparatus and an Eclipse Plus C8 (250 mm × 4.6 mm, 5 μm) analytical column were employed to perform the HPLC analysis of the crude extract and collected HSCCC effluents. Acetonitrile (A)-water (B) was assigned as the mobile phase, and the gradient elution was 0–5 min, 20–20% A; 5–10 min, 20–23% A; 10–25 min, 23–26%A; 26–40 min, 31–31%A; 41–60 min, 31–60%A; 61–70 min, 60–70%A; 71–80 min, 100–100%A, at a flow rate of 1.0 mL/min. The HPLC chromatograms were detected at 225 nm. All purified compounds were elucidated by analyzing the HRESI-MS and NMR spectroscopic data and comparing with data in literatures. 

## 4. Conclusions

In this present work, seven diterpene lactones, andrographolide (**1**), isoandrographolide (**2**), neo-andrographolide (**3**), 14-deoxy-11,12-didehydroandrographolide (**4**), 14-deoxyandrographiside (**5**), 14-deoxy-11,12-didehydroandrographiside (**6**), 3,14-dideoxyandrographolide (**10**), and three flavones, andrographidine C (**7**), andrographidine A (**8**), 5-hydroxy-7,8-dimethoxyflavanone (**9**) have been successfully and efficiently isolated from *A. paniculata* using an off-line 2D HSCCC method for the first time. Previously published papers about *A. paniculata* by HSCCC were mainly used the traditional unidimensional HSCCC, which usually led to the acquisition of a small number of purified compounds, such as andrographolide and neoandrographolide [13,18]. What is more, only one paper about *A. paniculata* was used for the off-line 2D HSCCC method [12], which rendered the isolation of five compounds. Compared with the above off-line 2D HSCCC method, this off-line 2D HSCCC method used here produced higher yields, is much more easily carried out, and more efficient, which not only has led to the isolation of the major compounds, but also the minor ones that appeared in the HPLC chromatograms of the extraction sample. Different hydrophilic solvent systems of petroleum ether-ethyl acetate-methanol-water were employed to run 1D and 2D HSCCC separations, which finally rendered ten compounds with wide range of polarities purified by the off-line 2D HSCCC method. The overall experiment results established that this off-line 2D HSCCC method improved not only the peak resolution of compounds with similar polarities but also purities and amounts of compounds with similar and different polarities. 

## Figures and Tables

**Table 1:** Partition coefficients (*K*_D_) of the target compounds in different solvent systems.

Solvent System	Ratio (*v*/*v*)	*K*_D_ Values of Target Compounds									
		1	2	3	4	5	6	7	8	9	10
petroleum ether-ethyl acetate-ethanol-water (*v*/*v*/*v*/*v*)	2:8:3:7	2.37	0.47	1.33	---	-	-	-	-	---	---
	2:8:5:5	1.87	0.15	3.57	---	0.23	0.96	-	---	---	---
petroleum ether-ethyl acetate-methanol-water	3:7:5:5	0.54	1.00	0.30	1.49	0.12	0.15	0.23	0.22	---	---
	3:7:4:6					-	-	-	-		
	3:7:3:7					-	-	-	-		
	2:8:1:9					0.30	0.50	1.20	0.78		
	5:5:6:4									0.30	0.80

‘---’ and ‘-’ stand for the K-values that were too large and too small, respectively.

## Supplementary Materials

Supplementary data (NMR and MS spectroscopic data for compounds **1**–**10**) associated with this article are available online, Figures S1–S30.

## References

[1] Committee of the Chinese Pharmacopia (2015). The Chinese Pharmacopia.

[2] Koteswara R.Y., Vimalamma G., Rao C.V., Tzeng Y.M. (2004). Flavonoids and andrographolides from *Andrographis paniculata*. Phytochemistry.

[3] Reddy M.K., Reddy M.V., Gunasekar D., Murthy M.M., Caux C., Bodo B. (2003). A flavone and an unusual 23-carbon terpenoid from *Andrographis paniculata*. Phytochemistry.

[4] Clander R., Srivastava V., Tandon J., Kapoor N.K. (1995). Antihepatotoxic activity of diterpenes of *Andrographis paniculata* (Kal-Megh) against Plasmodium berghei induced hepatic damage in Mastomys natalensis. Int. J. Pharmacog..

[5] Xia Y.F., Ye B.Q., Li Y.D., Wang J.G., He X.J., Lin X.F., Yao X.S., Ma D.W., Slungaard A., Hebbel R.P. (2004). Andrographolide attenuates inflammation by inhibition of NF-kappa B activation through covalent modification of reduced cysteine 62 of p50. J. Immunol..

[6] Parichatikanond W., Suthisisang C., Dhepakson P., Herunsalee A. (2010). Study of anti-inflammatory activities of the pure compounds from *Andrographis paniculata* (burm.f.) Nees and their effects on gene expression. Int. Immunopharmacol..

[7] Puri A., Saxena R., Saxena R.P., Saxena K.C., Srivastava V., Tandon J.S. (1993). Immounostimulant agents from *Andrographis paniculata*. J. Nat. Prod..

[8] Matsuda T., Kuroyangi M., Sugiyama S., Umehara K., Ueno A., Nishi K. (1994). Cell differentiation-inducing diterpenes from *Andrographis paniculata* Nees. Chem. Pharm. Bull..

[9] Liu Q., Shi S., Liu L., Yang H., Su W., Chen X. (2013). Separation and purification of bovine serum albumin binders from fructus polygoni orientalis using off-line two-dimensional complexation high-speed countercurrent chromatography target-guided by ligand fishing. J. Chromatogra. A.

[10] Yu J.Q., Zhao H.W., Wang D.J., Song X.Y., Zhao L., Wang X. (2017). Extraction and purification of five terpenoids from olibanum by ultrahigh pressure technique and high-speed countercurrent chromatography. J. Sep. Sci..

[11] Li H.L., Zhang Y.Q., Liu Q., Sun C.L., Li J., Yang P., Wang X. (2016). Preparative separation of phenolic compounds from Chimonanthus praecox Flowers by high-speed counter-current chromatography using a stepwise elution mode. Molecules.

[12] Zhang L., Liu Q., Yu J.G., Zeng H.L., Jiang S.J., Chen X.Q. (2015). Separation of five compounds from leaves of *Andrographis paniculata* (Burm. f.) Nees by off-line two-dimensional high-speed counter-current chromatography combined with gradient and recycling elution. J. Sep. Sci..

[13] Du Q.Z., Jerz G., Winterhalter P. (2003). S eparation of andrographolide and neoandrographolide from the leaves of *Andrographis paniculata* using high-speed counter-current chromatography. J. Chromatogr. A.

[14] Fujita T., Fujitani R., Takeda Y., Takaishi Y., Yamada T., Kido M., Miura I. (1984). On the diterpenoids of *Andrographis paniculata*: X-ray crystallographic analysis of andrographolide and structure determination of new minor diterpenoids. Chem. Pharm. Bull..

[15] Balmain A., Connolly J.D. (1973). Minor diterpenoids constituents of *Andrographis paniculata*. Nees. J. Chem. Soc. Perkin Trans. I.

[16] Chen W.M., Liang X.T. (1982). Deoandrographolide-19β-D-Glucoside from the leaves of *Andrographis paniculata*. Planta Med..

[17] Kuroyanagi M., Sato M., Ueno A. (1987). Flavonoids from *Andrographis paniculata*. Chem. Pharm. Bull..

[18] Zhang Y.Q., Wang S.S. (2017). Online hyphenation of extraction, Sephadex LH-20 column chromatography, and high-speed counter-current chromatography: A highly efficient strategy for the preparative separation of andrographolide from *Andrographis paniculata* in a single step. J. Sep. Sci..

