# Supplementary material for: Preparative Separation of Diterpene Lactones and Flavones from Andrographis paniculate Using Off-Line Two-Dimensional High-Speed Counter-Current Chromatography
Source: Molecules. 2019 Feb 11;24(3):620. doi: 10.3390/molecules24030620 (PMC6384559; doi:10.3390/molecules24030620)
Supplement: Supplementary file 1 [file molecules-24-00620-s001.pdf]

Preparative Separation of diterpene lactones and flavones from *Andrographis paniculata* by off-line two-dimentional High-Speed Counter-Current Chromatography

Xiaowei Sun[1,2], Huijiao Yan[1], Yujie Zhang[3], Xiao Wang[1,2*], Dawei Qin[2*] and Jinqian Yu [1*]

[1] Shandong Key Laboratory of TCM Quality Control Technology, Shandong Analysis and Test Center, Qilu University of Technology (Shandong Academy of Sciences), Jinan 250014, China; sxw199301@163.com (X.S.); yanhuijiao01@163.com (H.Y.); wangx@sdas.org (X.W.); yujinqian87528@126.com (J.Y.)

[2] School of Chemistry and Pharmaceutical Engineering, Qilu University of Technology (Shandong Academy of Sciences), Jinan 250014, China; sxw199301@163.com (X.S.); wangx@sdas.org (X.W.); qdw109@163.com (D.Q.)

[3] School of Life Sciences, Linyi University, Linyi 276000, China; zhangyujie@lyu.edu.cn (Y.Z.);

Supplementary material

Table of Contents

**no.**	Content	Page
**1**	**Figure S1.** The HREIMS Spectroscopic Data of Compound **1**	S4
**2**	**Figure S2.** The ^{1}H NMR Spectrum of Compound **1** in pyridine-d_5 (400 MHz)	S5
**3**	**Figure S3.** The ^{13}C NMR Spectrum of Compound **1** in pyridine-d_5 (100 MHz)	S6
**4**	**Figure S4.** The HREIMS Spectroscopic Data of Compound **2**	S7
**5**	**Figure S5.** The ^{1}H NMR Spectrum of Compound **2** in pyridine-d_5 (400 MHz)	S8
**6**	**Figure S6.** The ^{13}C NMR Spectrum of Compound **2** in pyridine-d_5 (100 MHz)	S9
**7**	**Figure S7.** The HREIMS Spectroscopic Data of Compound **3**	S10
**8**	**Figure S8.** The ^{1}H NMR Spectrum of Compound **3** in pyridine-d_5 (400 MHz)	S11
**9**	**Figure S9.** The ^{13}C NMR Spectrum of Compound **3** in pyridine-d_5 (100 MHz)	S12
**10**	**Figure S10.** The HREIMS Spectroscopic Data of Compound **4**	S13
**11**	**Figure S11.** The ^{1}H NMR Spectrum of Compound **4** in pyridine-d_5 (400 MHz)	S14
**12**	**Figure S12.** The ^{13}C NMR Spectrum of Compound **4** in pyridine-d_5 (100 MHz)	S15
**13**	**Figure S13.** The HREIMS Spectroscopic Data of Compound **5**	S16
**14**	**Figure S14.** The ^{1}H NMR Spectrum of Compound **5** in pyridine-d_5 (400 MHz)	S17
**15**	**Figure S15.** The ^{13}C NMR Spectrum of Compound **5** in pyridine-d_5 (100 MHz)	S18
**16**	**Figure S16.** The HREIMS Spectroscopic Data of Compound **6**	S19
**17**	**Figure S17.** The ^{1}H NMR Spectrum of Compound **6** in pyridine-d_5 (400 MHz)	S20
**18**	**Figure S18.** The ^{13}C NMR Spectrum of Compound **6** in pyridine-d_5 (100 MHz)	S21
**19**	**Figure S19.** The HREIMS Spectroscopic Data of Compound **7**	S22

**20**	**Figure S20.** The ^{1}H NMR Spectrum of Compound **7** in pyridine-d_5 (400 MHz)	S23
**21**	**Figure S21.** The ^{13}C NMR Spectrum of Compound **7** in pyridine-d_5 (100 MHz)	S24
**22**	**Figure S22.** The HREIMS Spectroscopic Data of Compound **8**	S25
**23**	**Figure S23.** The ^{1}H NMR Spectrum of Compound **8** in pyridine-d_5 (400 MHz)	S26
**24**	**Figure S24.** The ^{13}C NMR Spectrum of Compound **8** in pyridine-d_5 (100 MHz)	S27
**25**	**Figure S25.** The HREIMS Spectroscopic Data of Compound **9**	S28
**26**	**Figure S26.** The ^{1}H NMR Spectrum of Compound **9** in pyridine-d_5 (400 MHz)	S29
**27**	**Figure S27.** The ^{13}C NMR Spectrum of Compound **9** in pyridine-d_5 (100 MHz)	S30
**28**	**Figure S28.** The HREIMS Spectroscopic Data of Compound **10**	S31
**29**	**Figure S29.** The ^{1}H NMR Spectrum of Compound **10** in pyridine-d_5 (400 MHz)	S32
**30**	**Figure S30.** The ^{13}C NMR Spectrum of Compound **10** in pyridine-d_5 (100 MHz)	S33

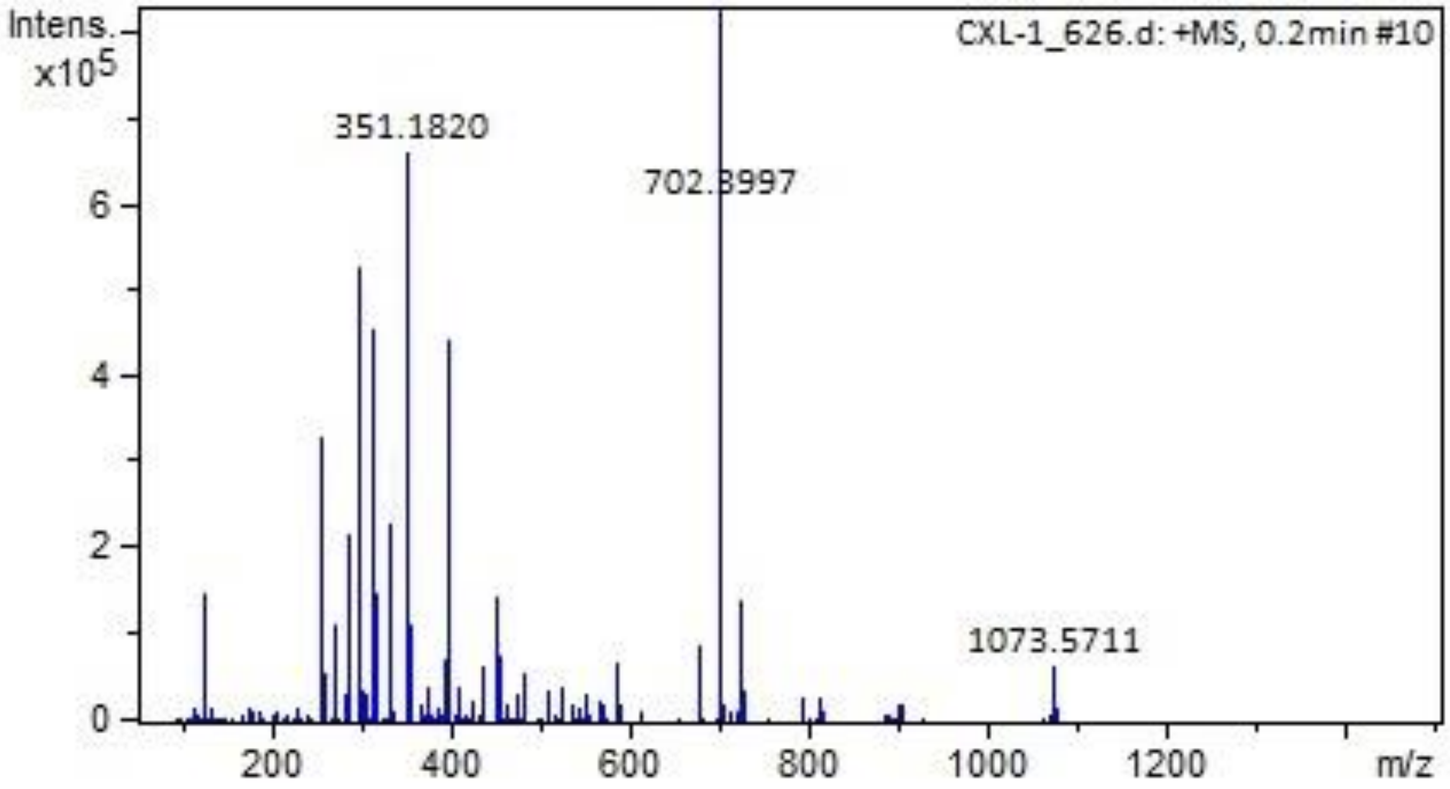

Figure S1. The HREIMS Spectroscopic Data of Compound **1**

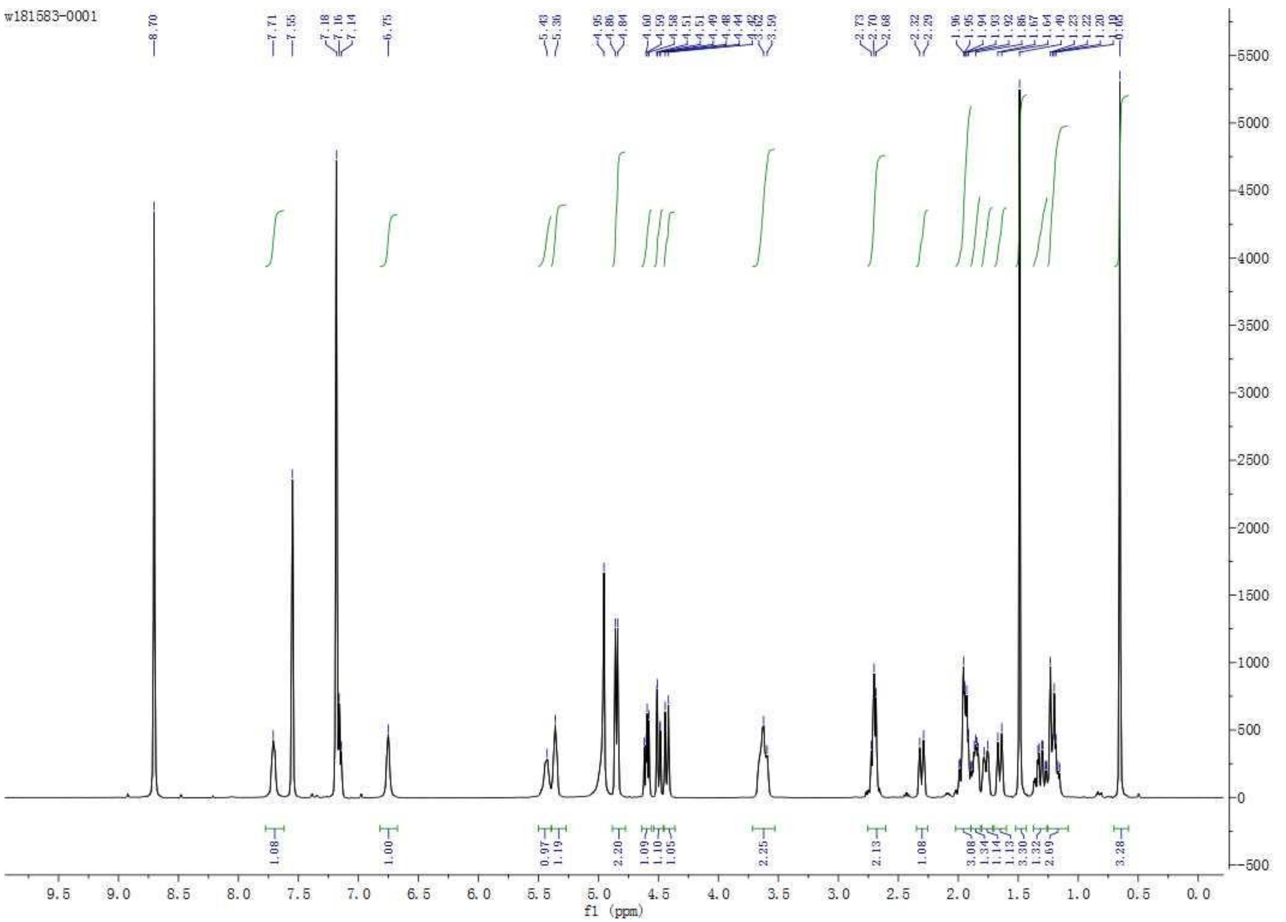

Figure S2. The ^{1}H NMR Spectrum of Compound **1** in pyridine-d_5 (400 MHz)

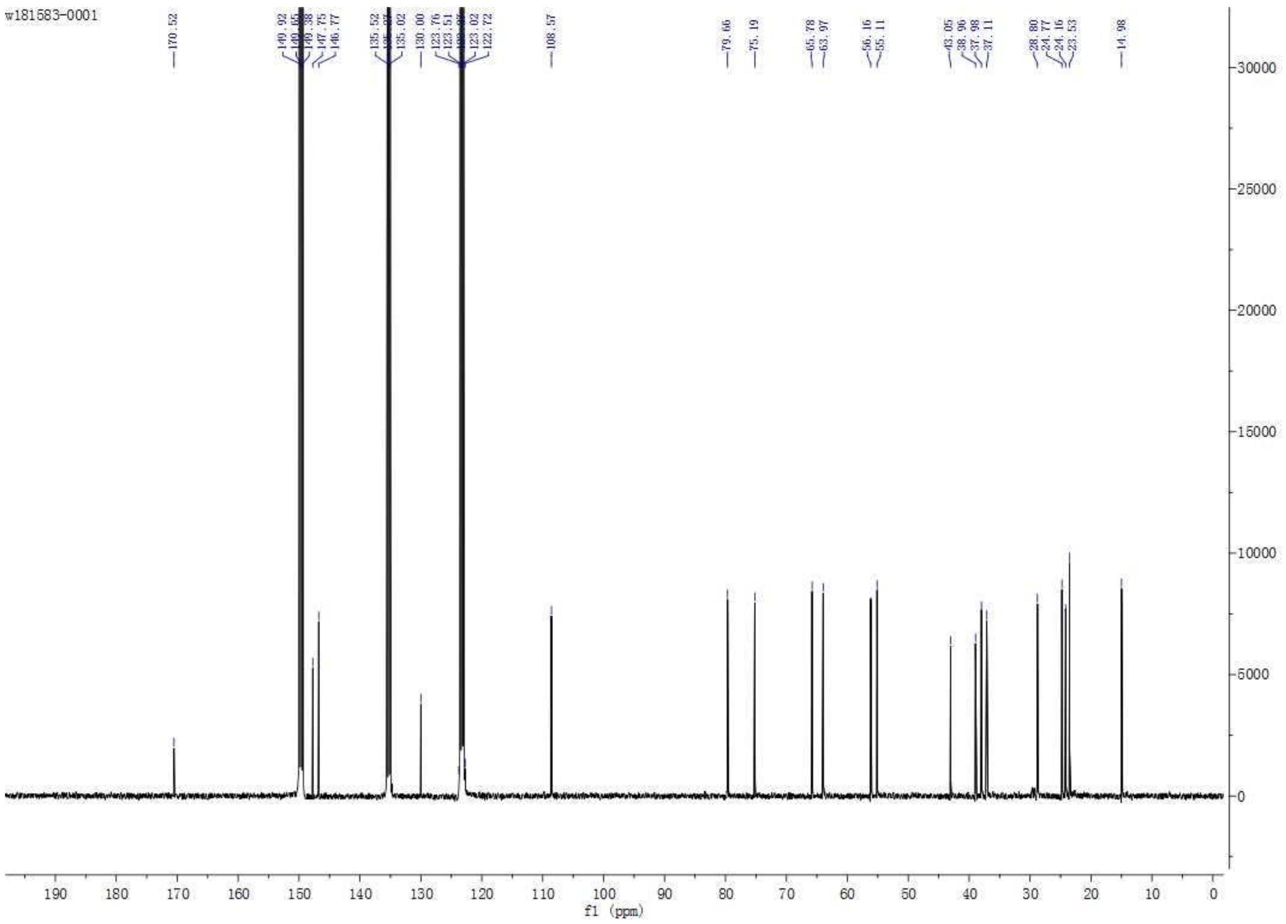

Figure S3. The ^{13}C NMR Spectrum of Compound **1** in pyridine-d_5 (100 MHz)

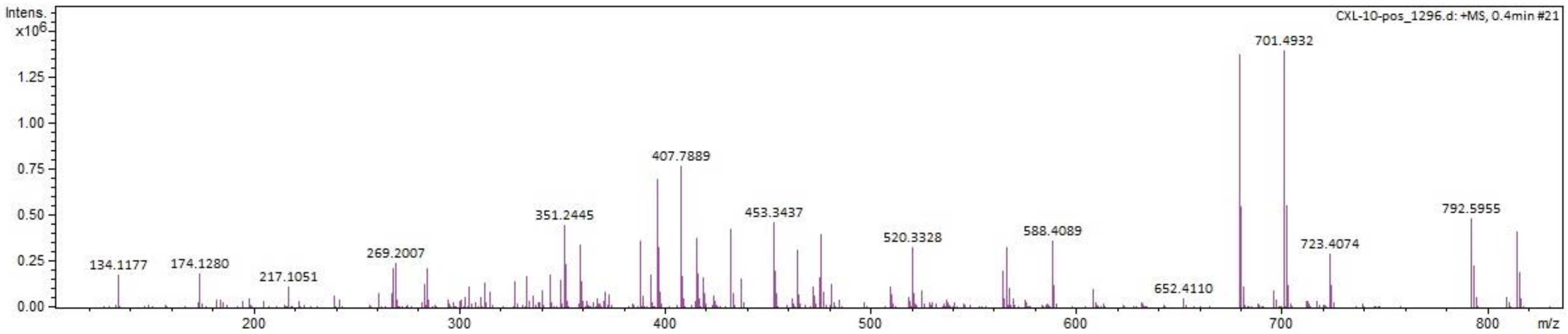

Figure S4. The HREIMS Spectroscopic Data of Compound **2**

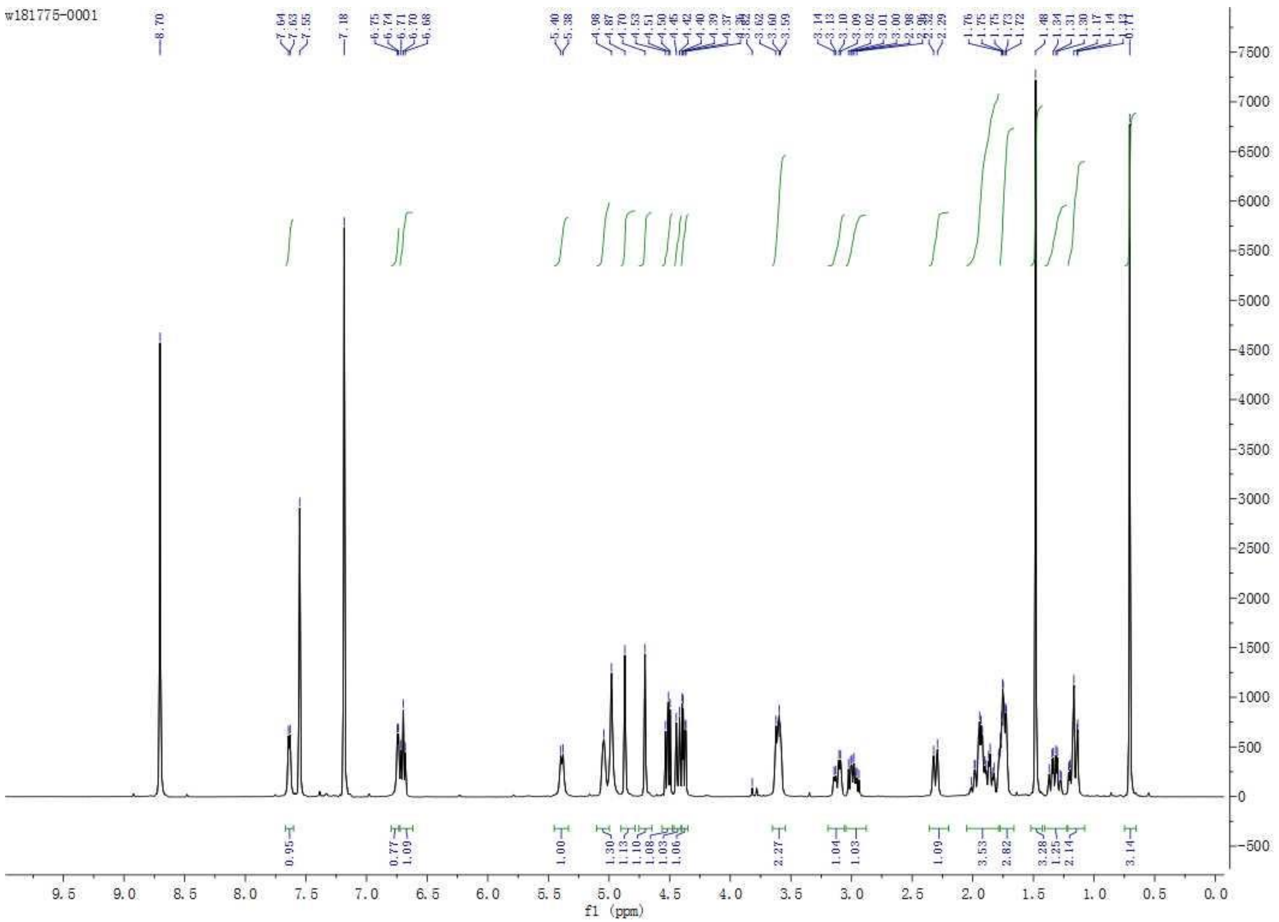

Figure S5. The ^{1}H NMR Spectrum of Compound **2** in pyridine-d_5 (400 MHz)

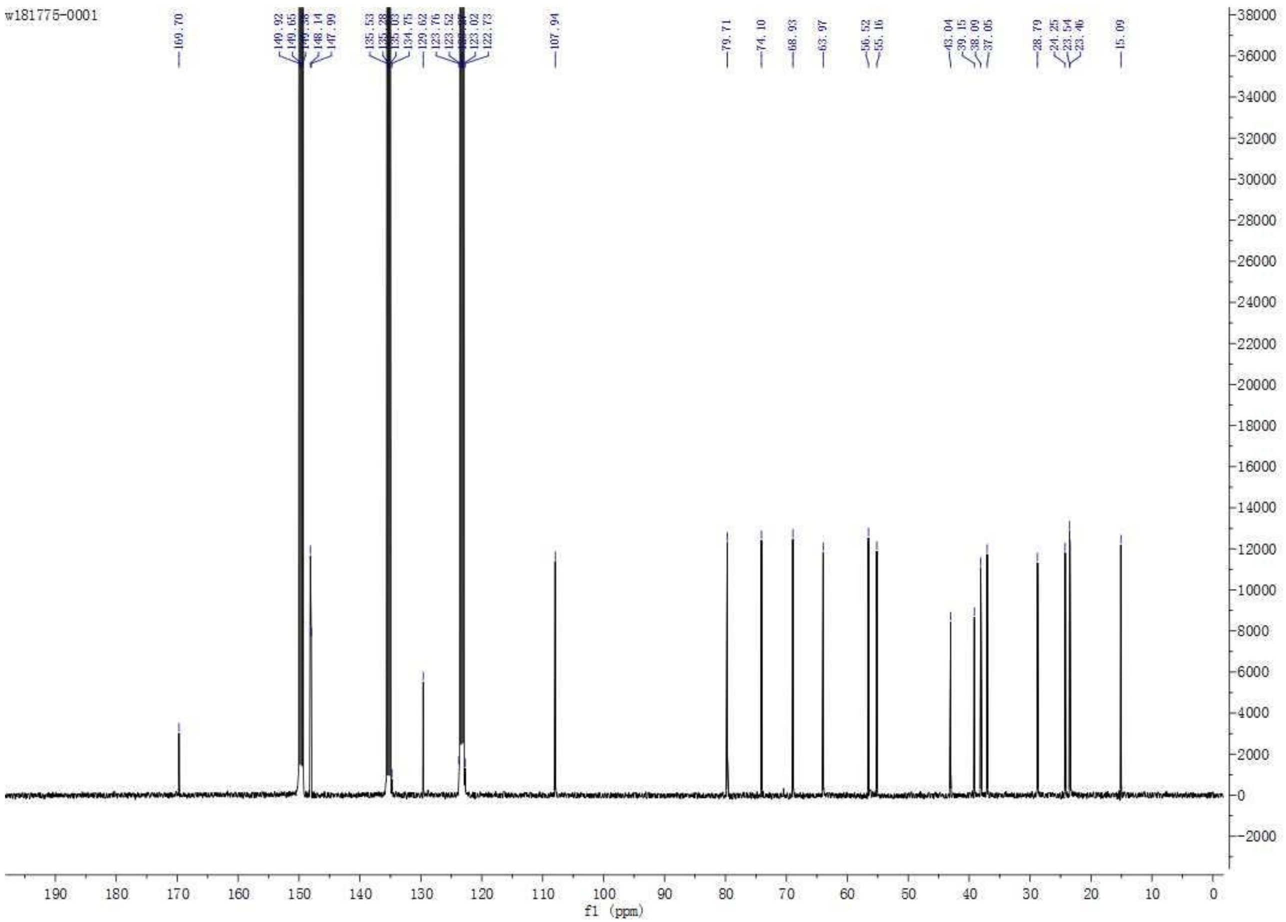

Figure S6. The ^{13}C NMR Spectrum of Compound **2** in pyridine-d_5 (100 MHz)

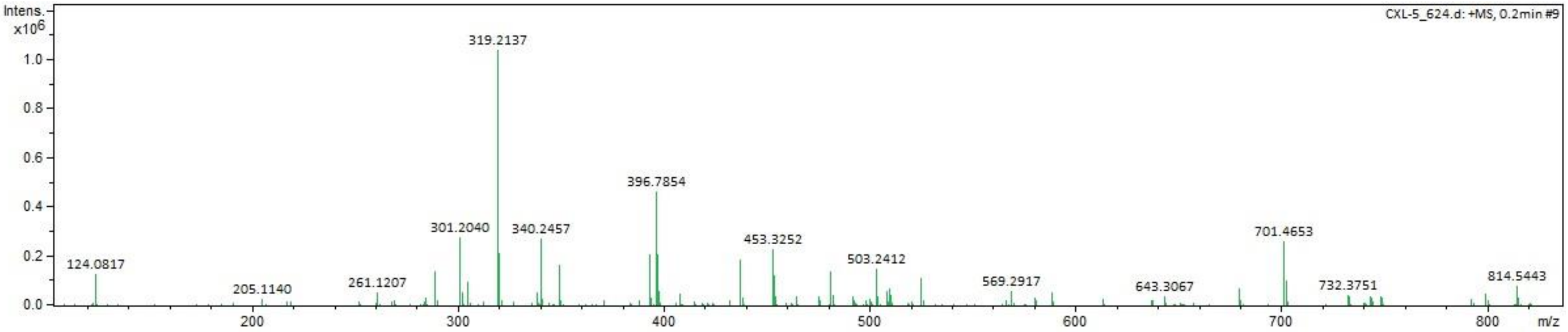

Figure S7. The HREIMS Spectroscopic Data of Compound **3**

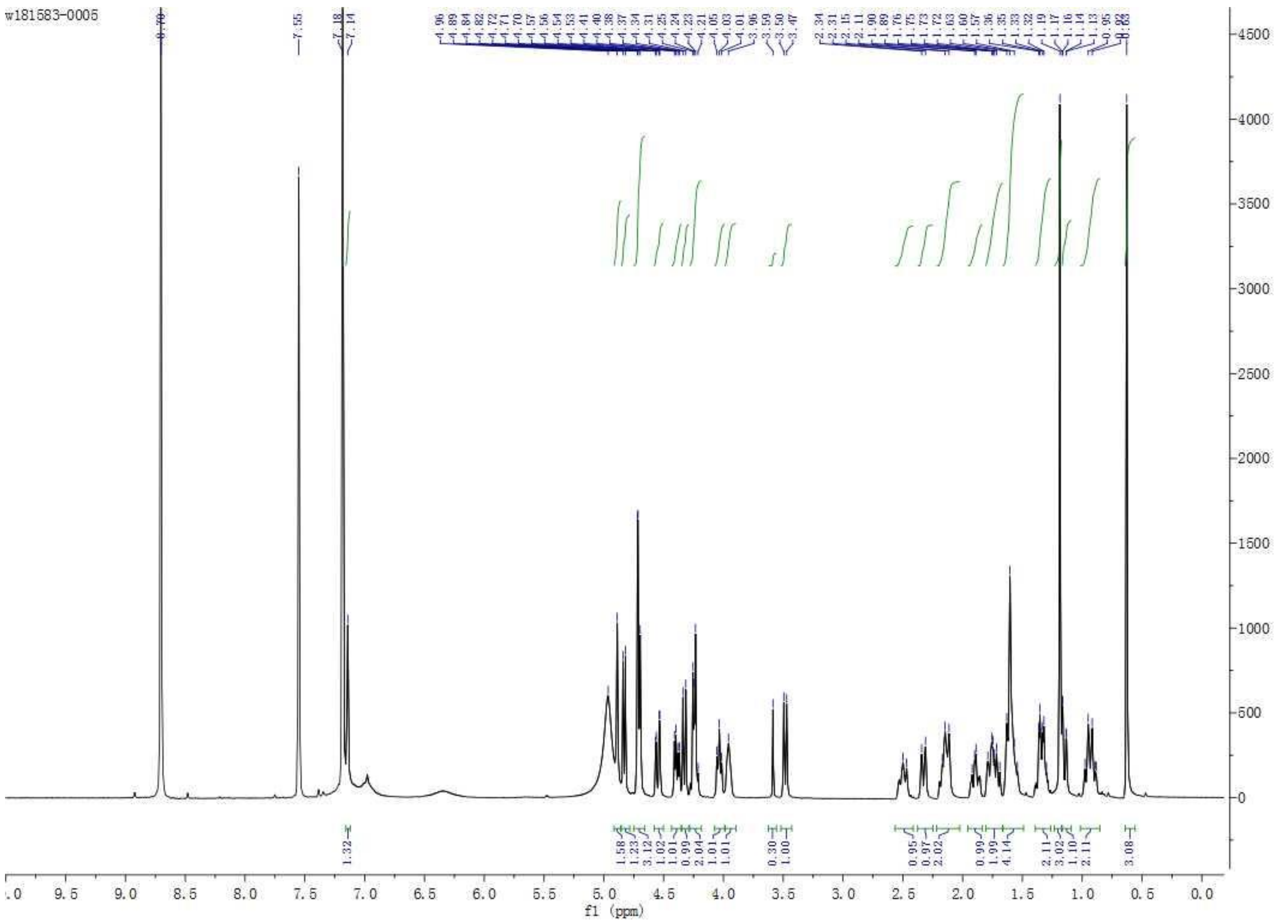

Figure S8. The ^{1}H NMR Spectrum of Compound **3** in pyridine-d_5 (400 MHz)

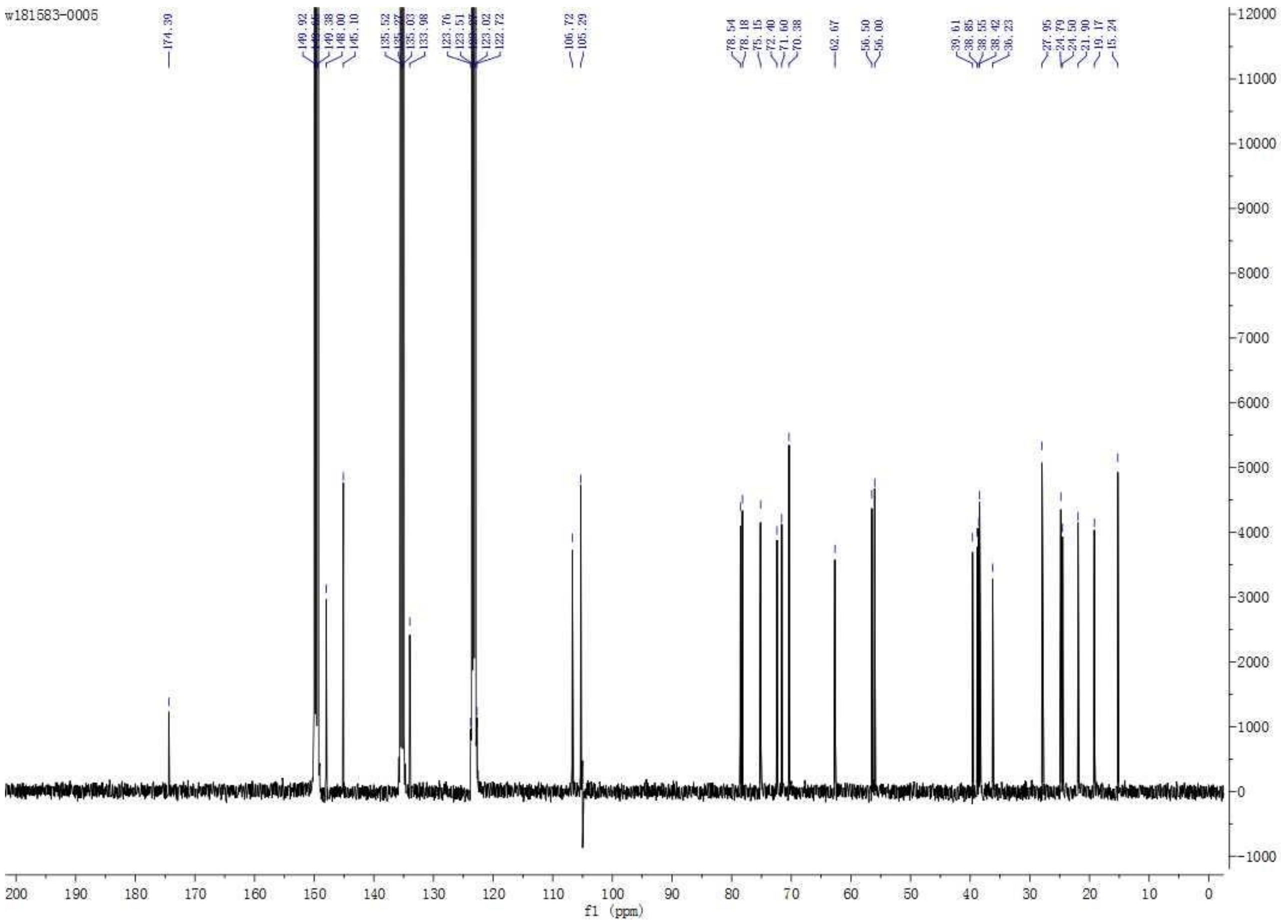

Figure S9. The ^{13}C NMR Spectrum of Compound **3** in pyridine-d_5 (100 MHz)

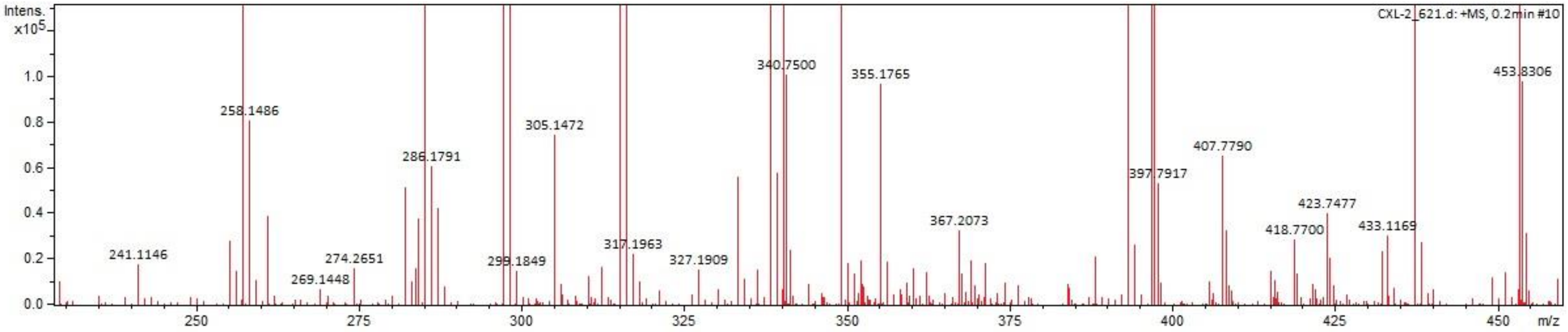

Figure S10. The HREIMS Spectroscopic Data of Compound **4**

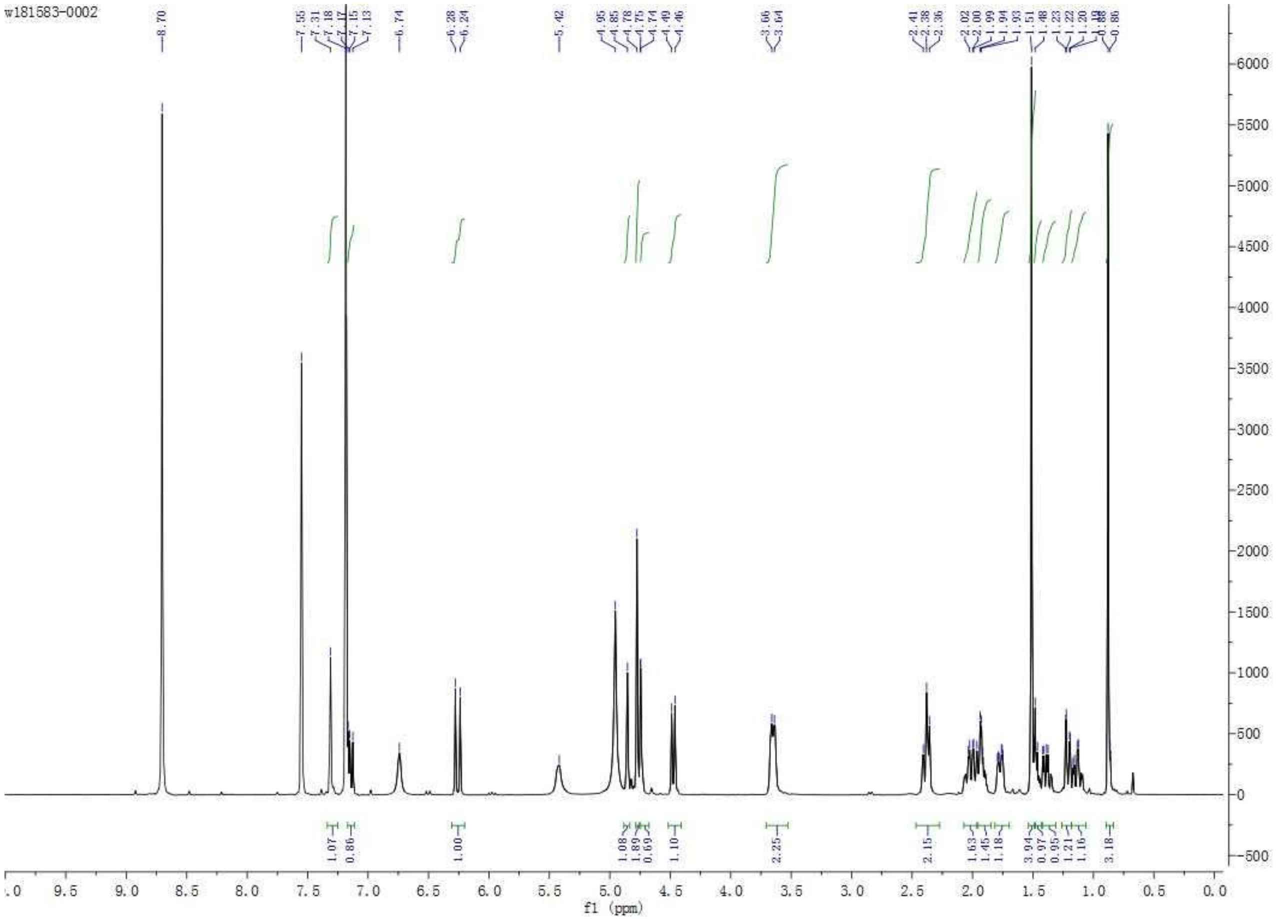

Figure S11. The ^{1}H NMR Spectrum of Compound **4** in pyridine-d_5 (400 MHz)

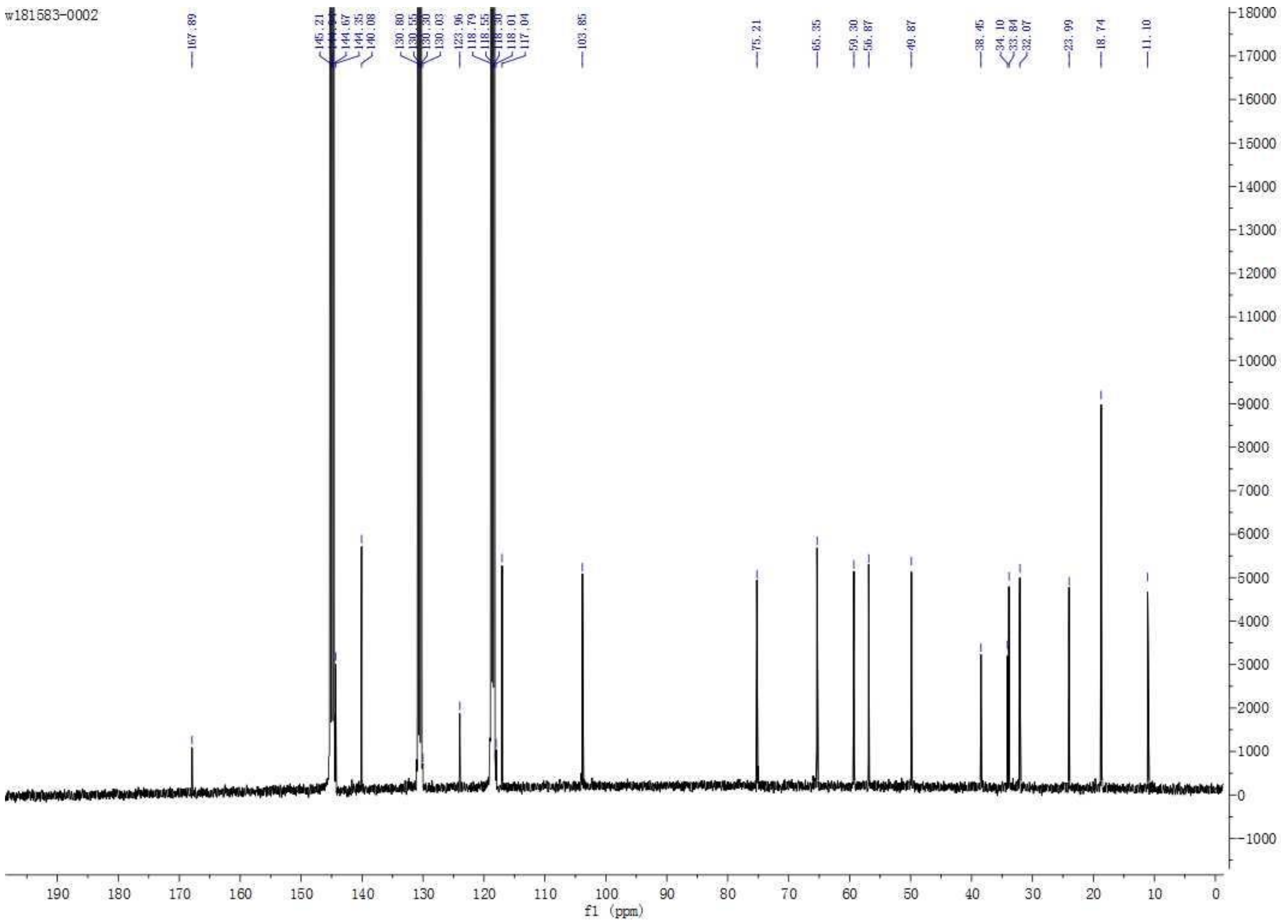

Figure S12. The ^{13}C NMR Spectrum of Compound **4** in pyridine-d_5 (100 MHz)

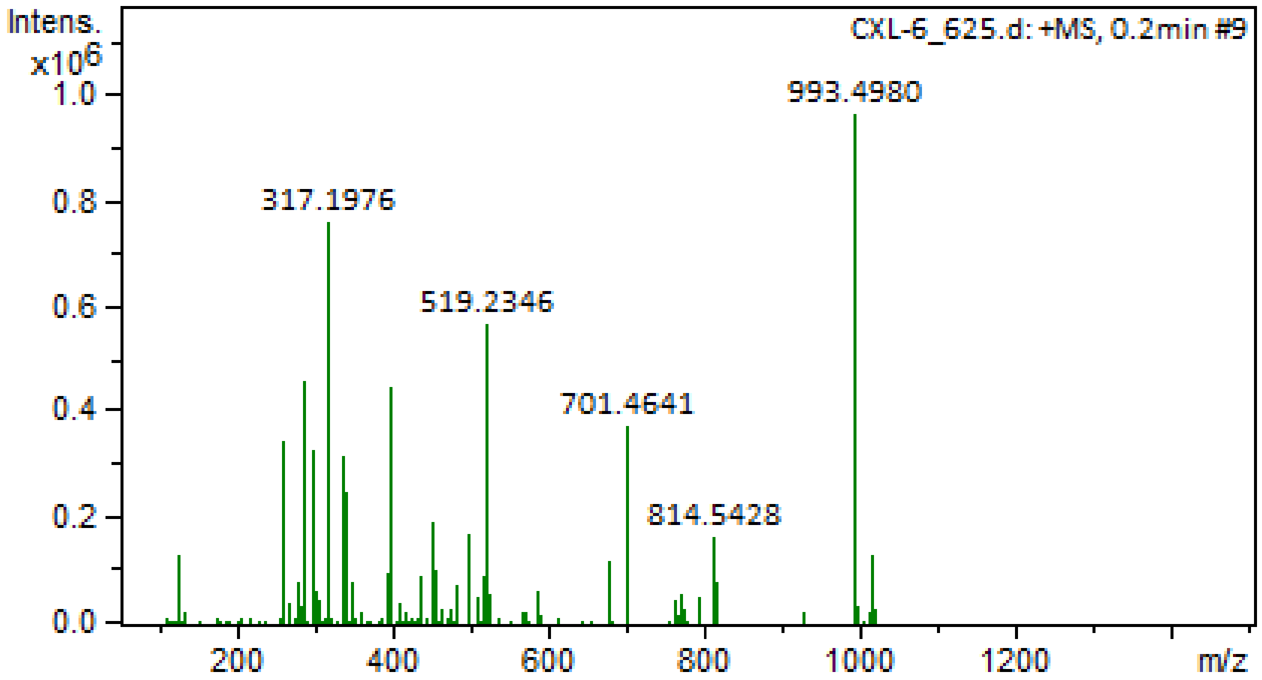

Figure S13. The HREIMS Spectroscopic Data of Compound **5**

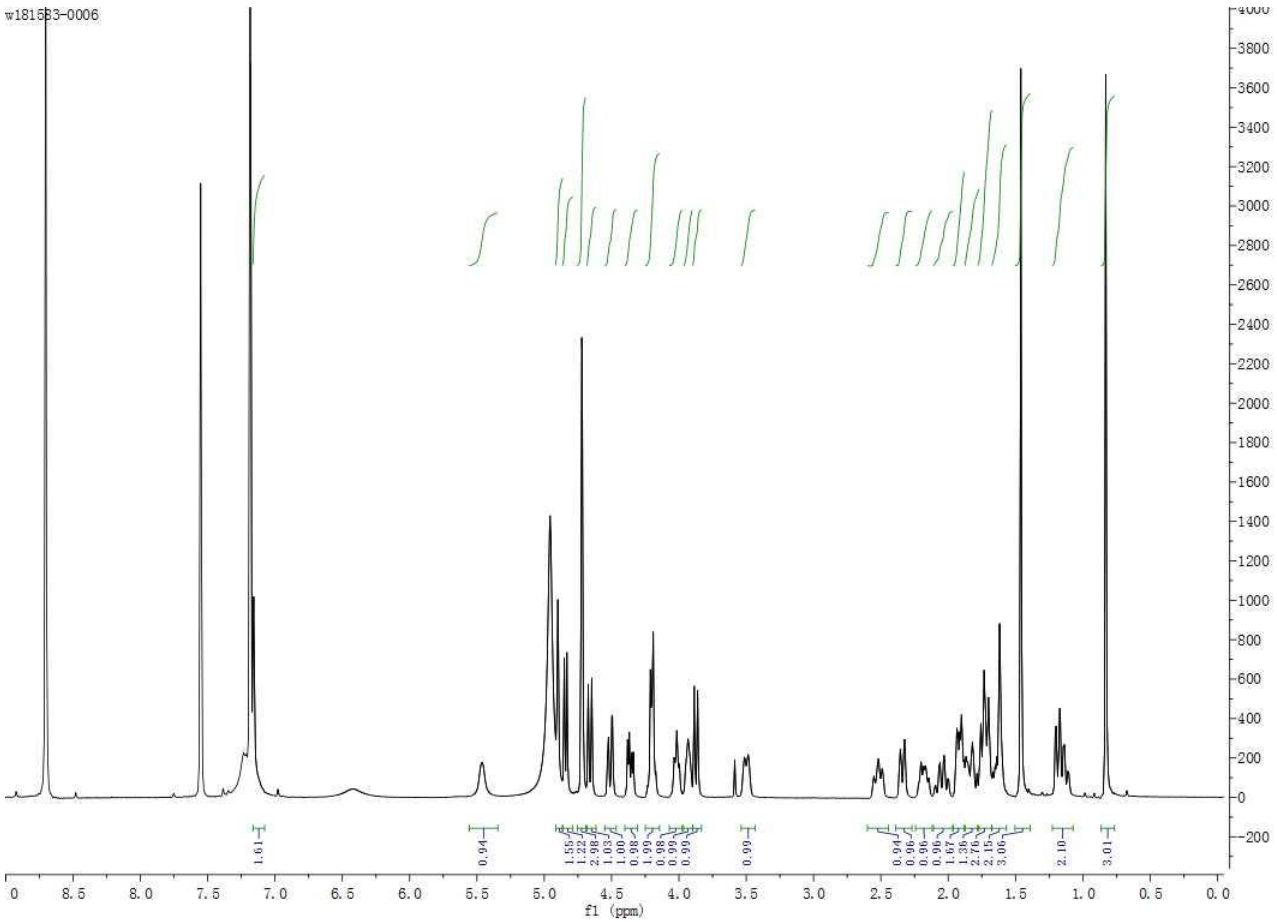

Figure S14. The ^{1}H NMR Spectrum of Compound **5** in pyridine-d_5 (400 MHz)

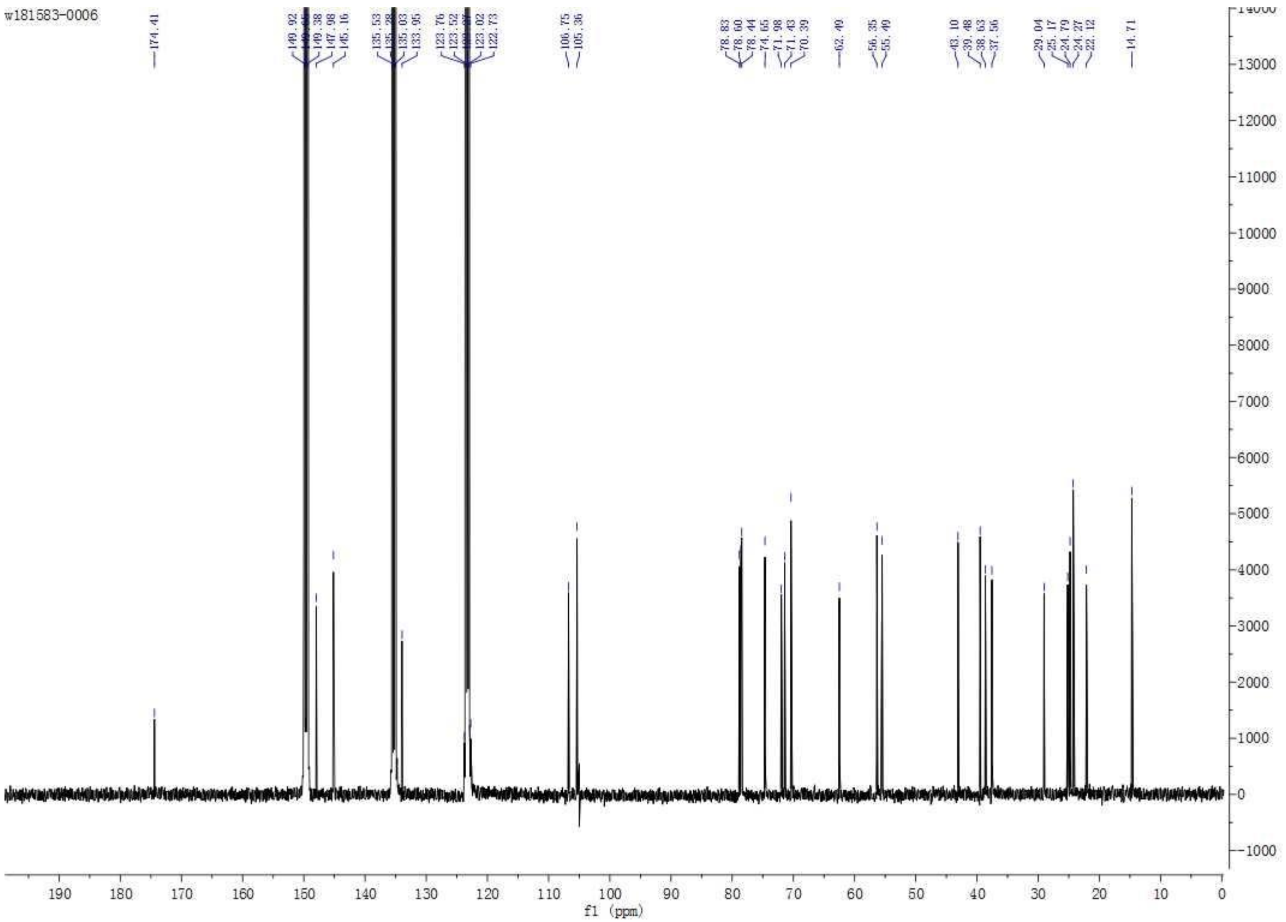

Figure S15. The ^{13}C NMR Spectrum of Compound **5** in pyridine-d_5 (100 MHz)

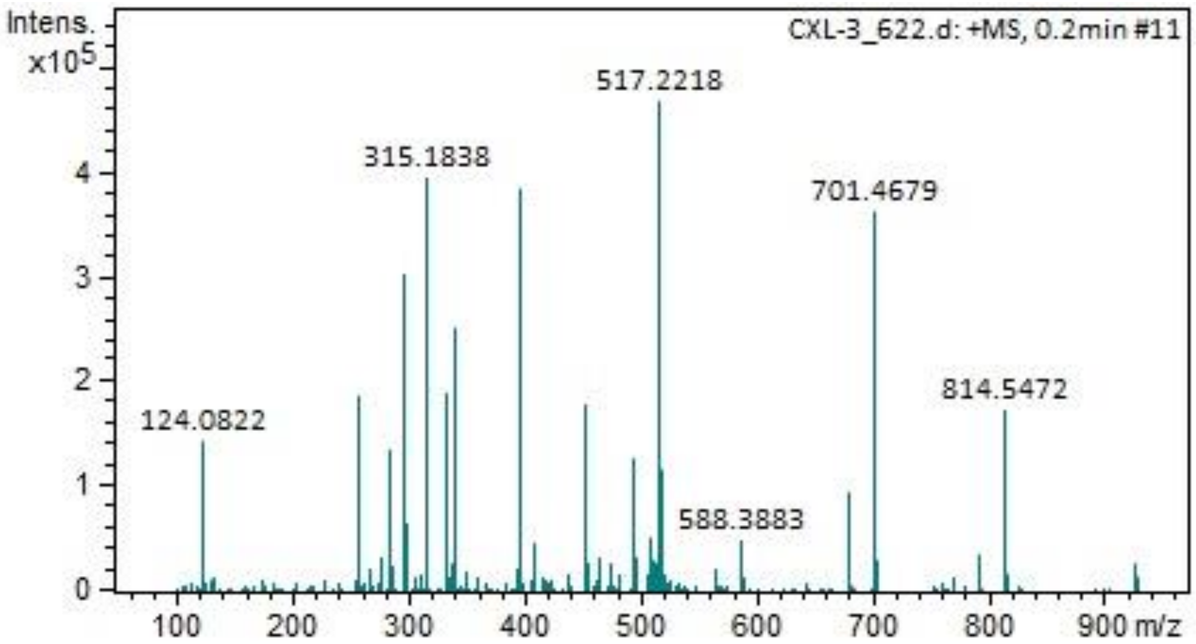

Figure S16. The HREIMS Spectroscopic Data of Compound **6**

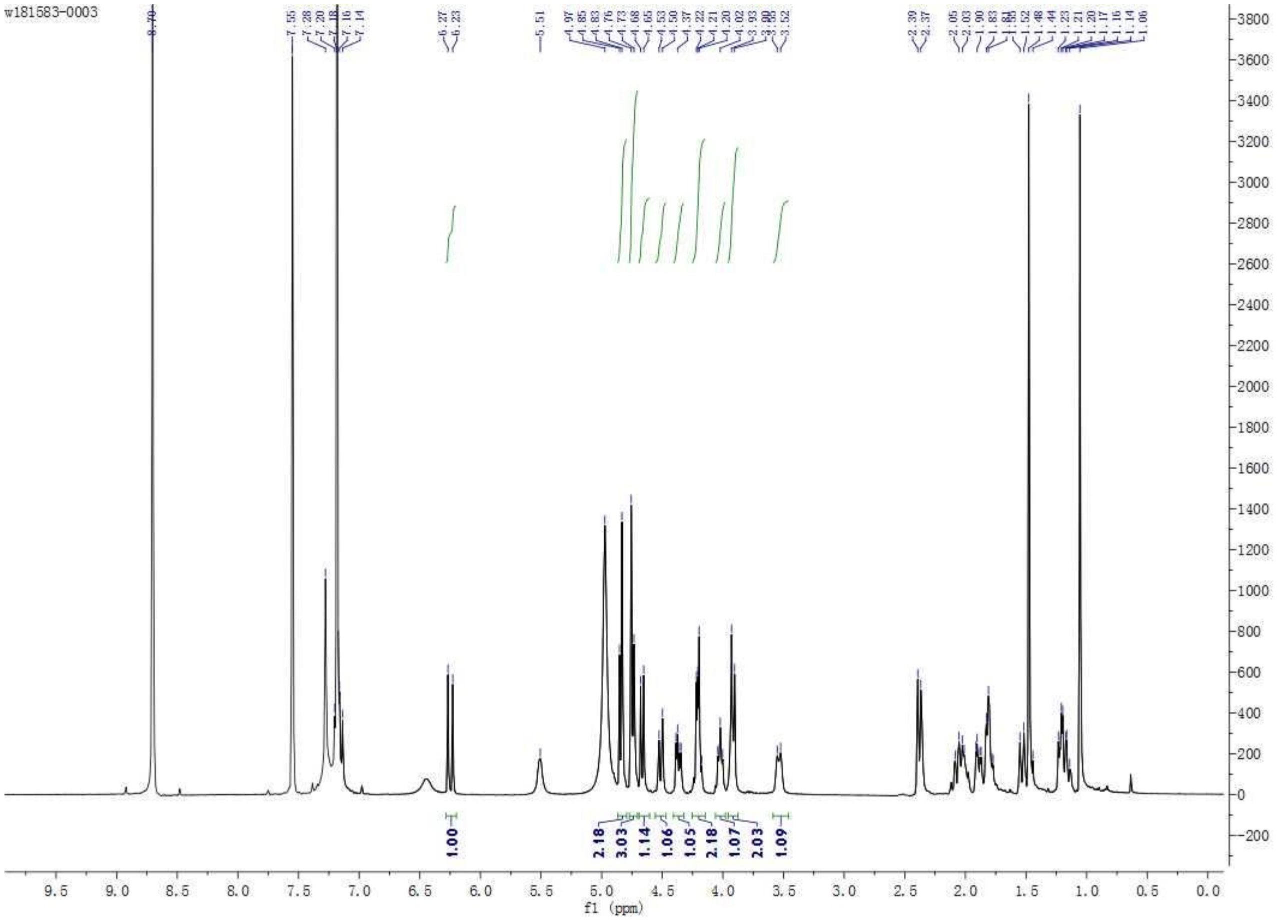

Figure S17. The ^{1}H NMR Spectrum of Compound **6** in pyridine-d_5 (400 MHz)

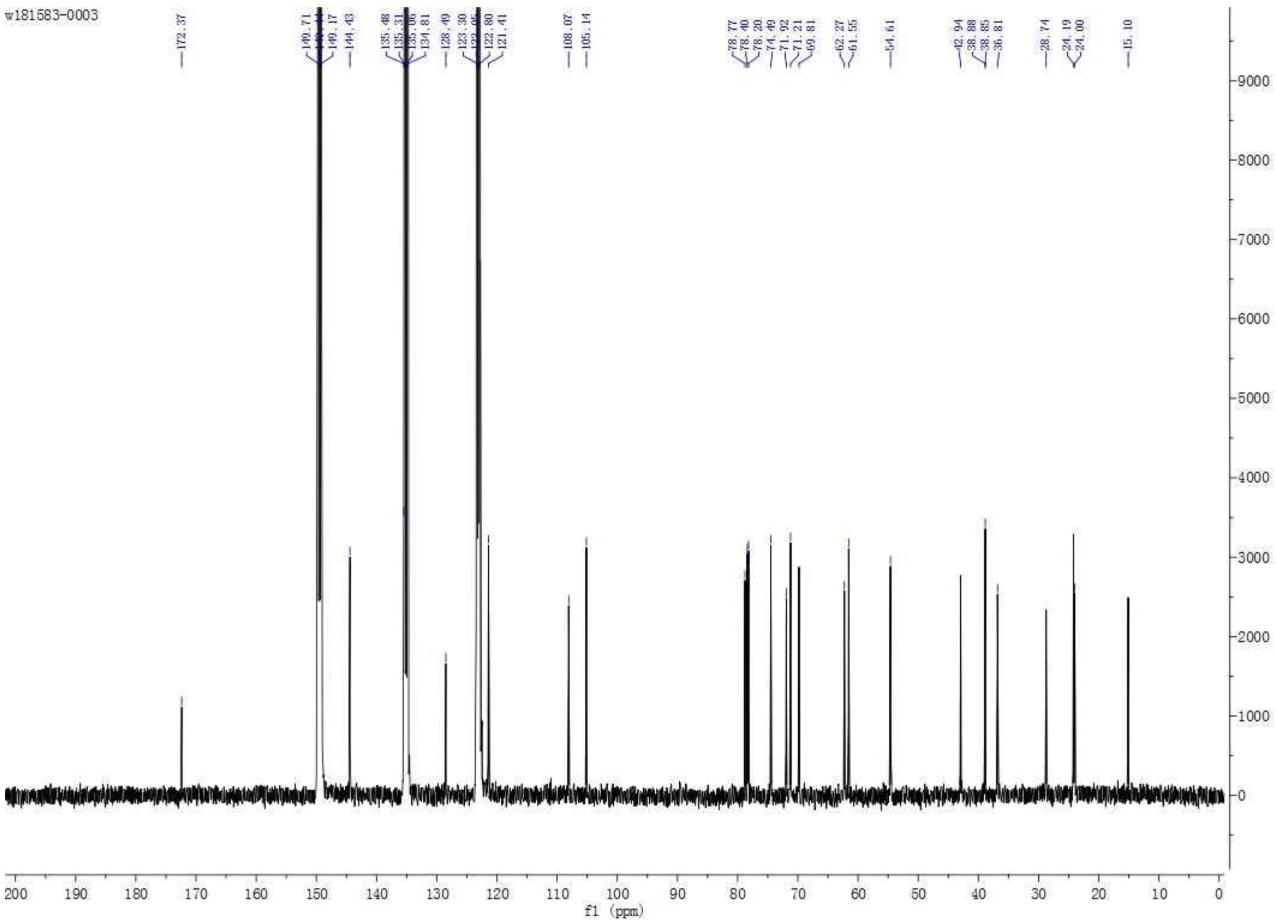

Figure S18. The ^{13}C NMR Spectrum of Compound **6** in pyridine-d_5 (100 MHz)

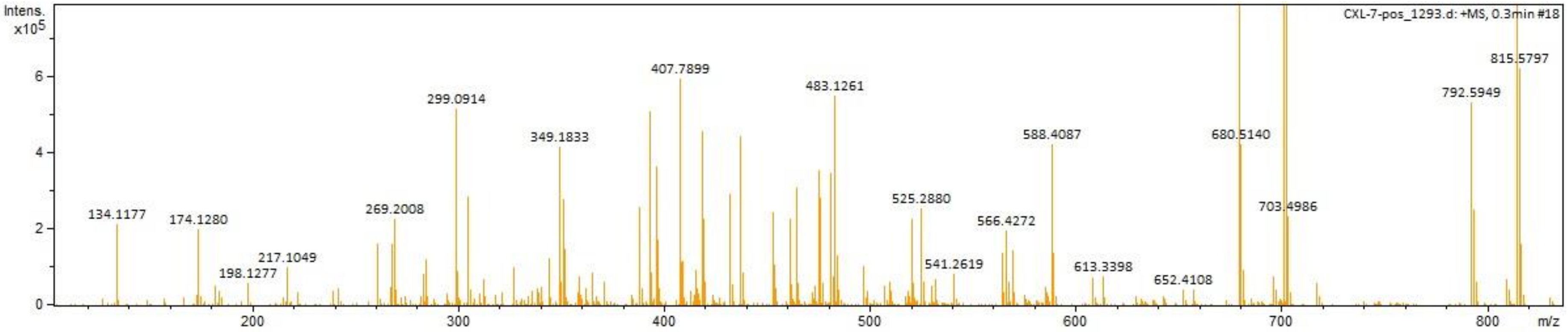

Figure S19. The HREIMS Spectroscopic Data of Compound **7**

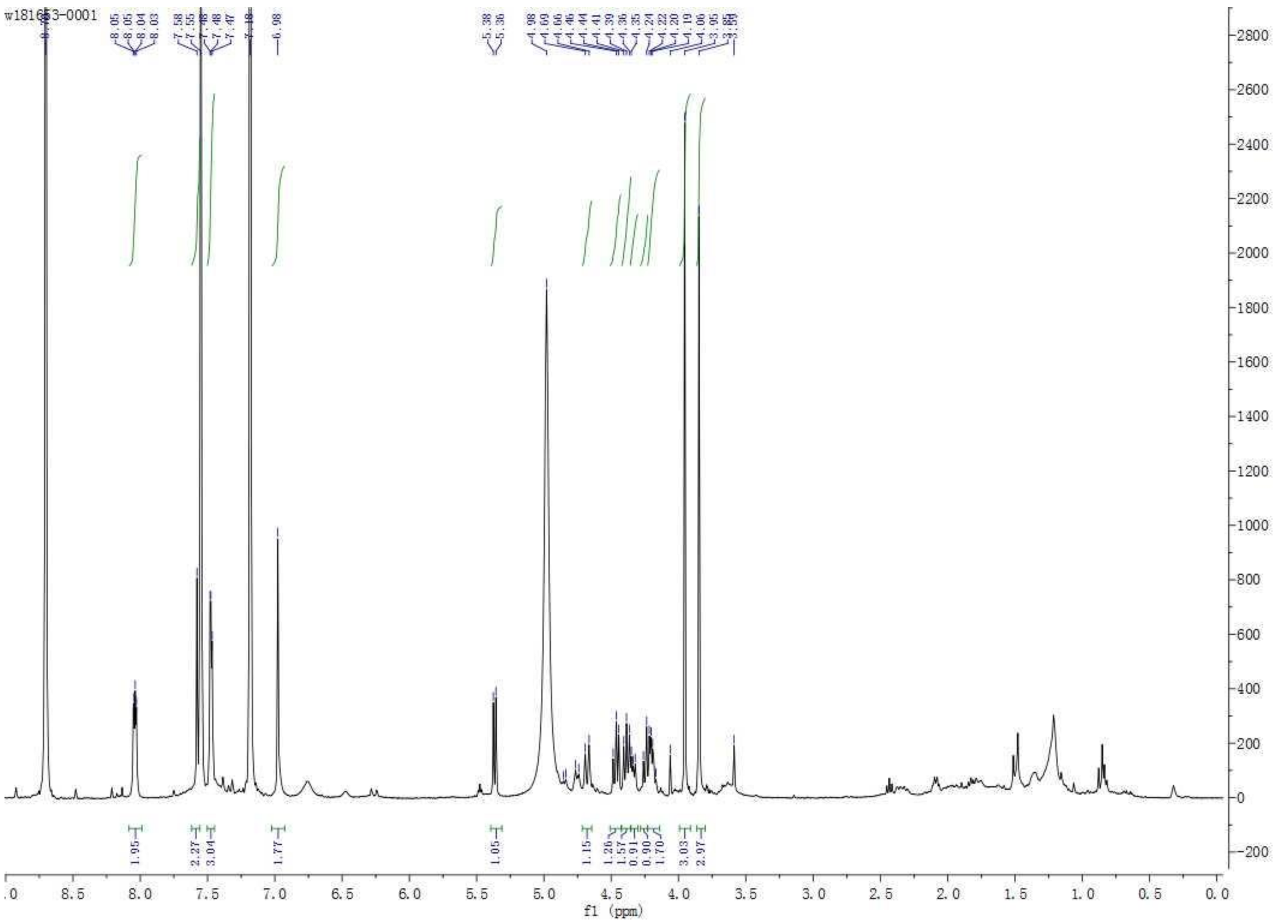

Figure S20. The ^{1}H NMR Spectrum of Compound **7** in pyridine-d_5 (400 MHz)

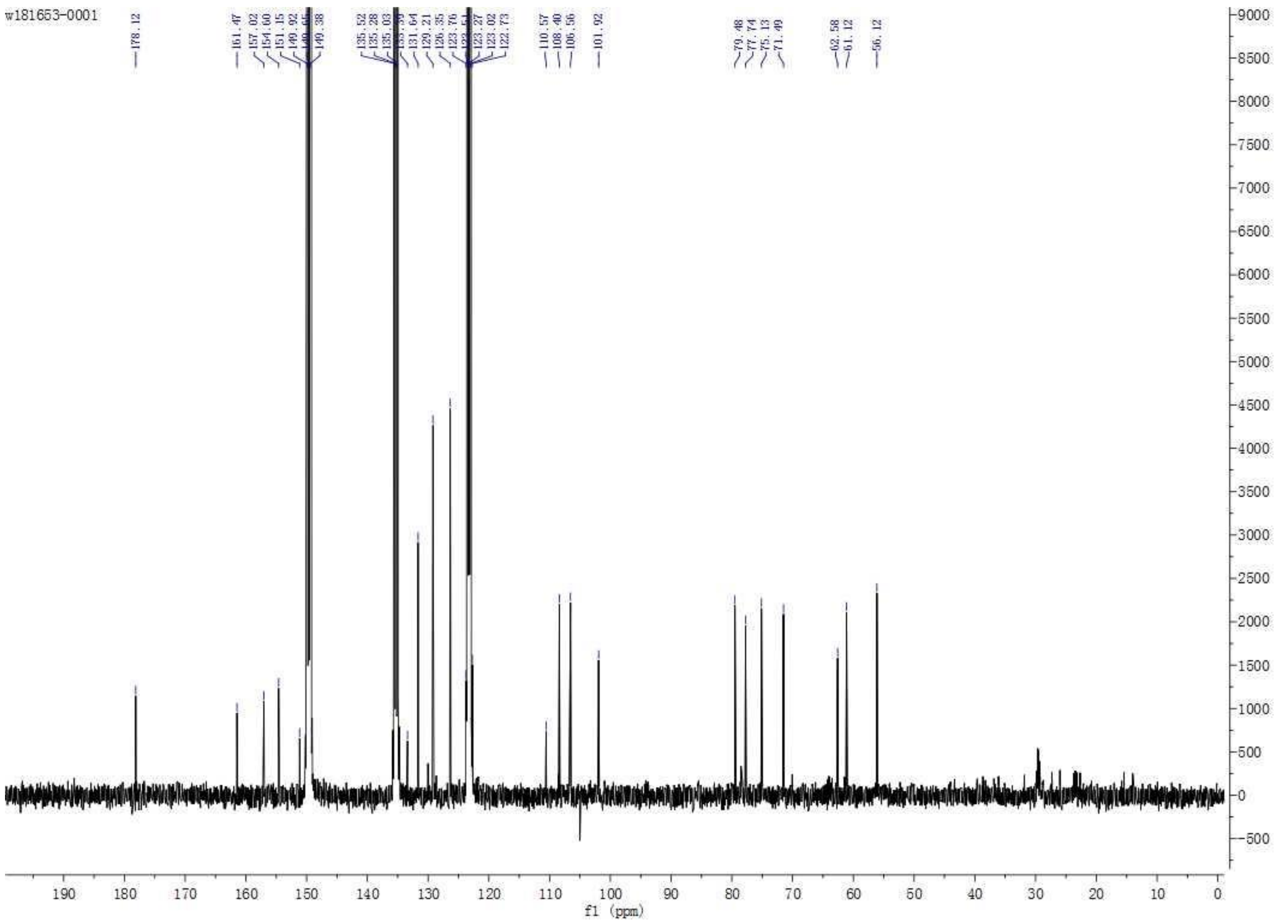

Figure S21. The ^{13}C NMR Spectrum of Compound **7** in pyridine-d_5 (100 MHz)

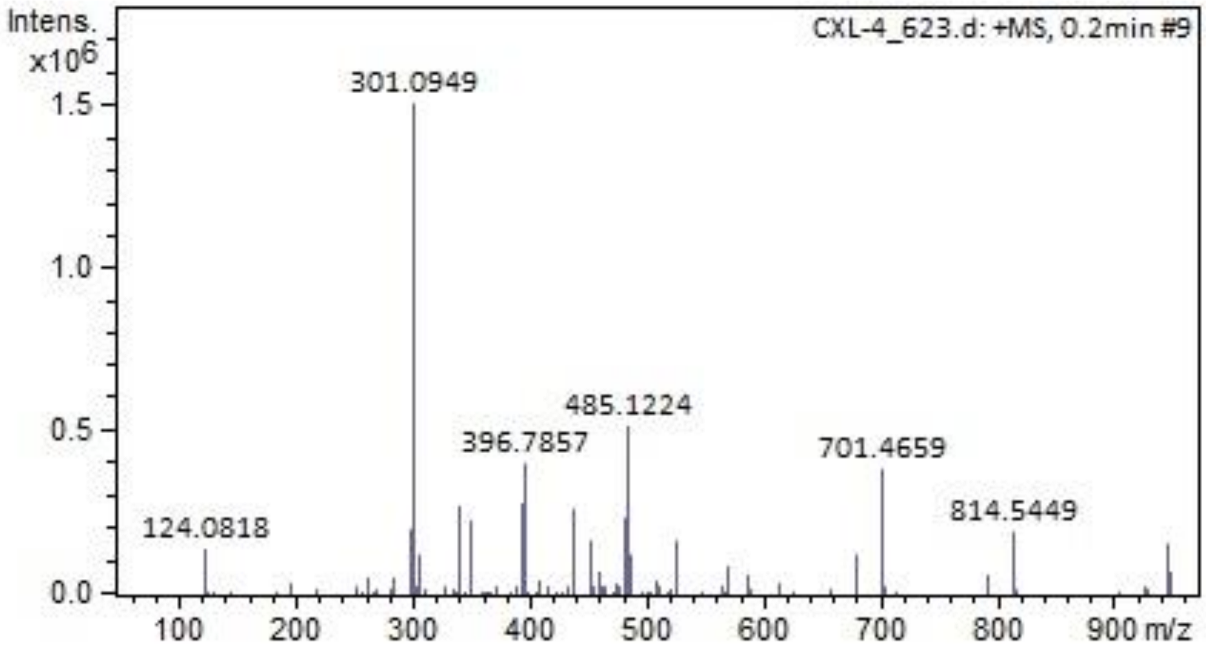

Figure S22. The HREIMS Spectroscopic Data of Compound **8**

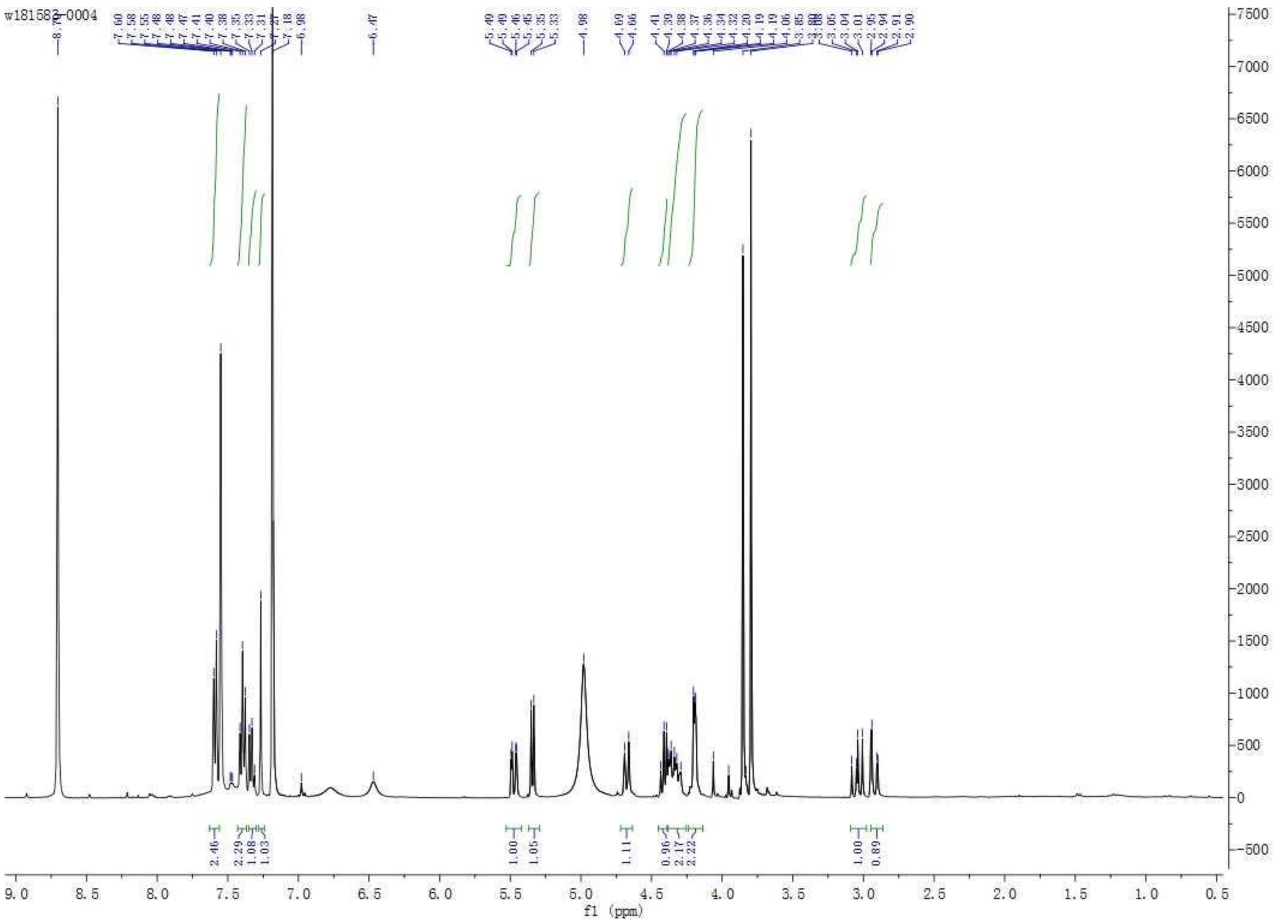

Figure S23. The ^{1}H NMR Spectrum of Compound **8** in pyridine-d_5 (400 MHz)

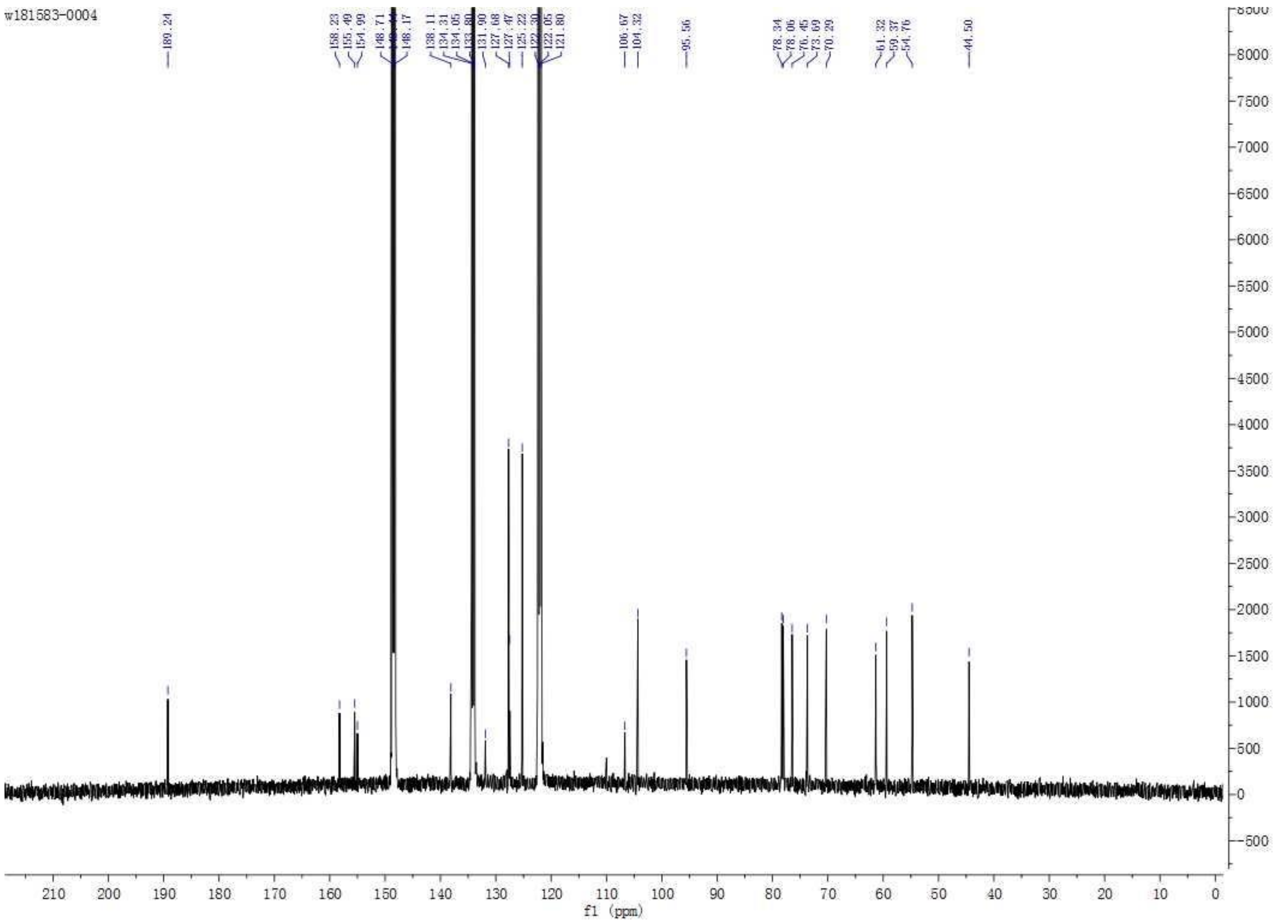

Figure S24. The ^{13}C NMR Spectrum of Compound **8** in pyridine-d_5 (100 MHz)

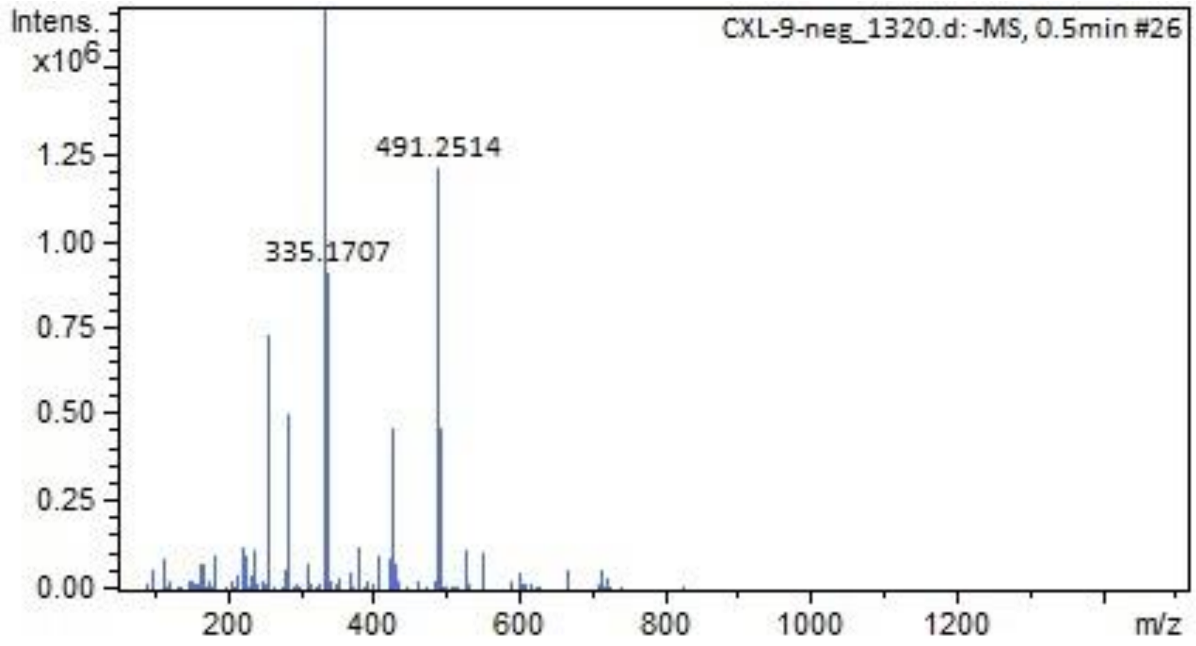

Figure S25. The HREIMS Spectroscopic Data of Compound **9**

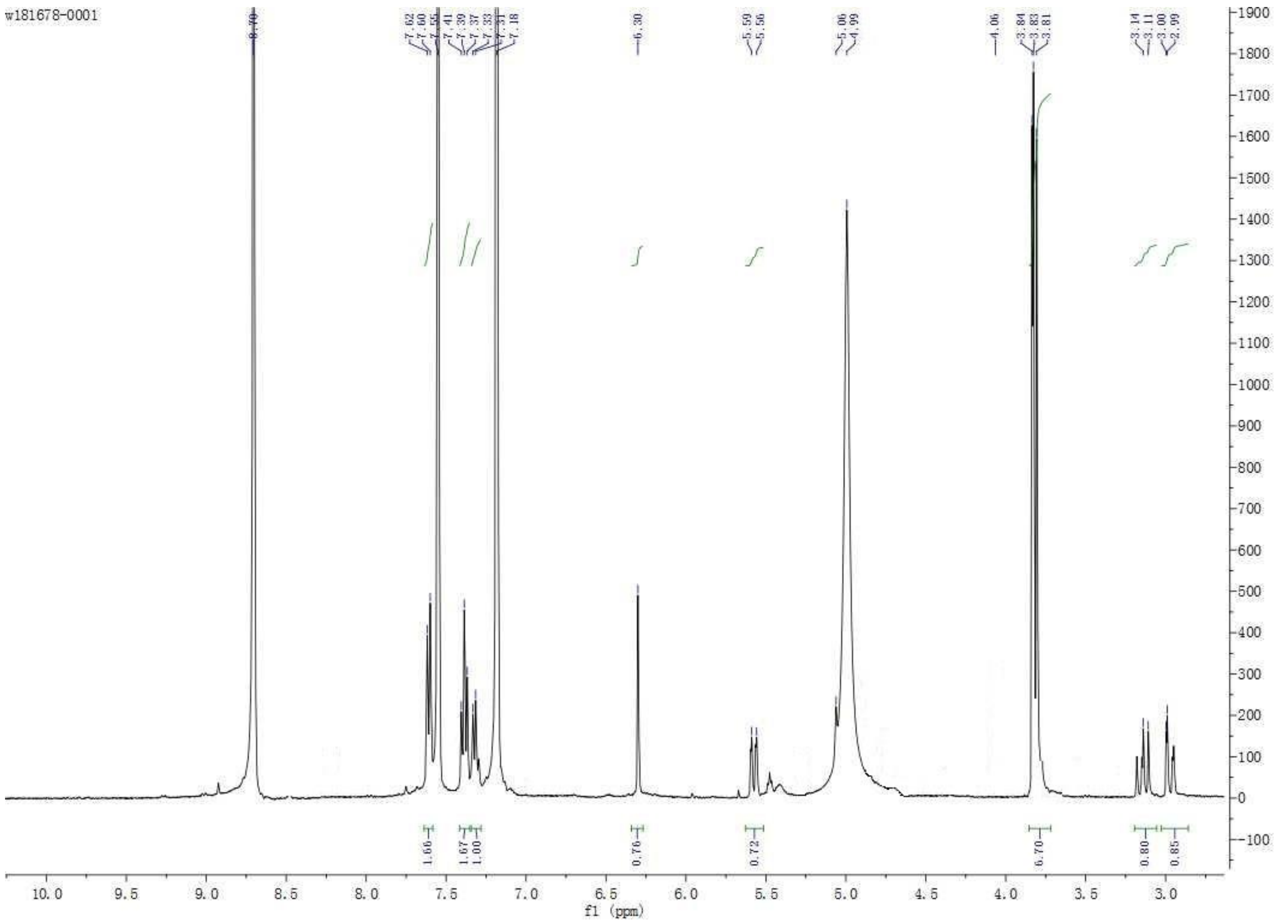

Figure S26. The ^{1}H NMR Spectrum of Compound **9** in pyridine-d_5 (400 MHz)

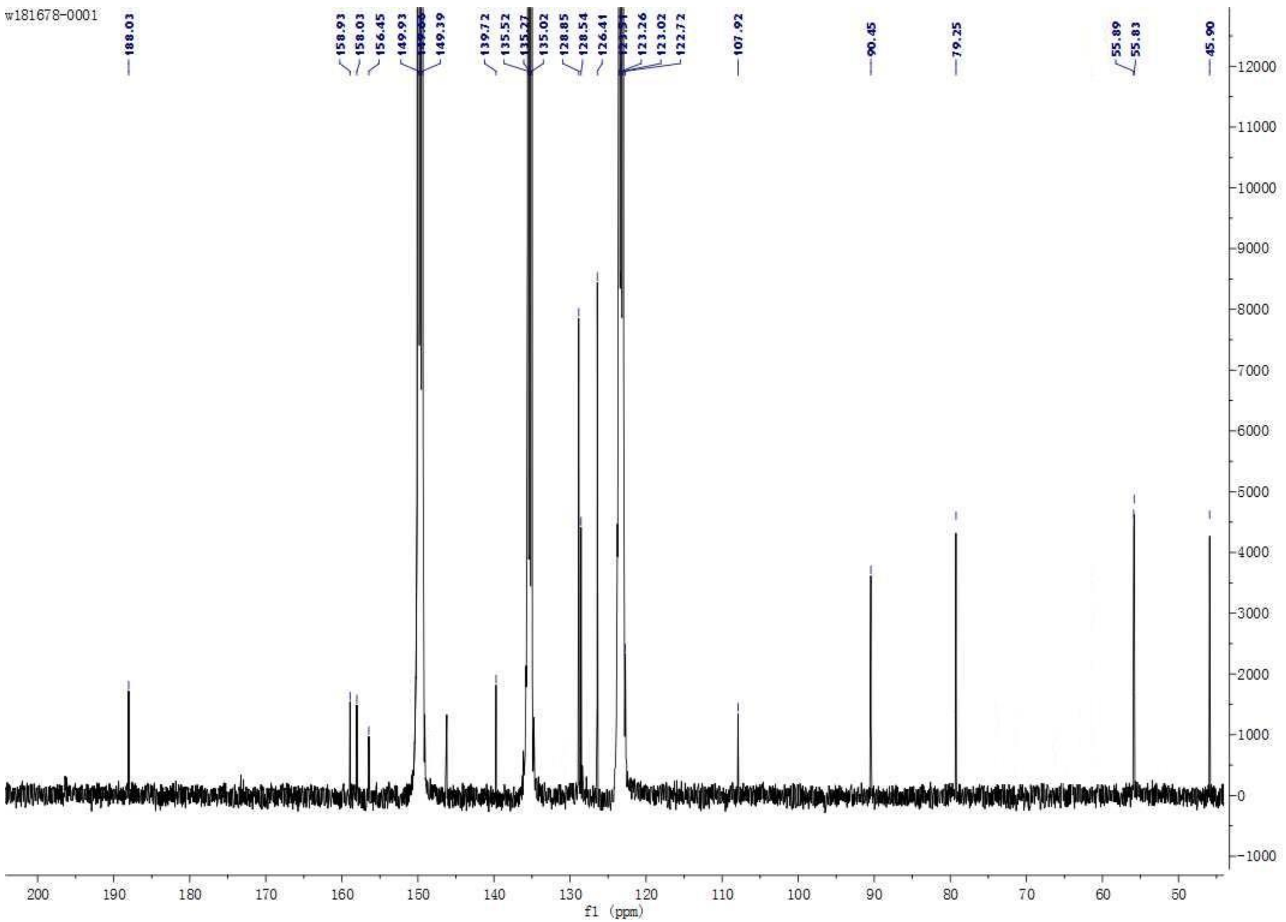

Figure S27. The ^{13}C NMR Spectrum of Compound **9** in pyridine-d_5 (100 MHz)

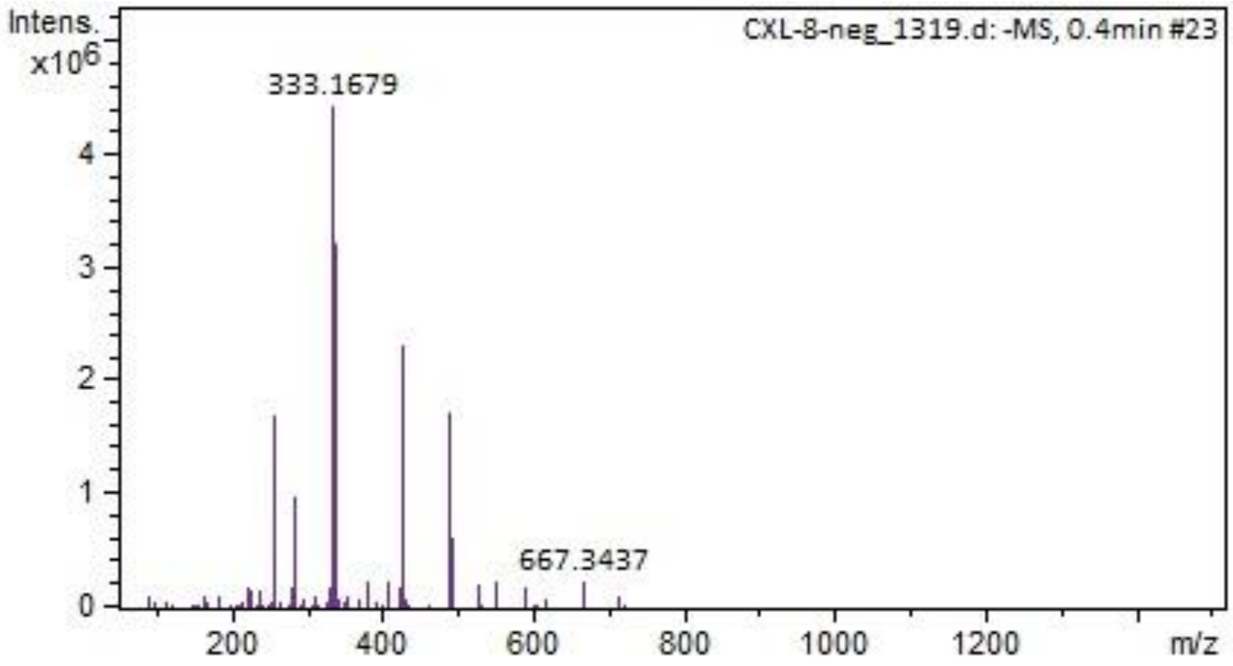

Figure S28. The HREIMS Spectroscopic Data of Compound **10**

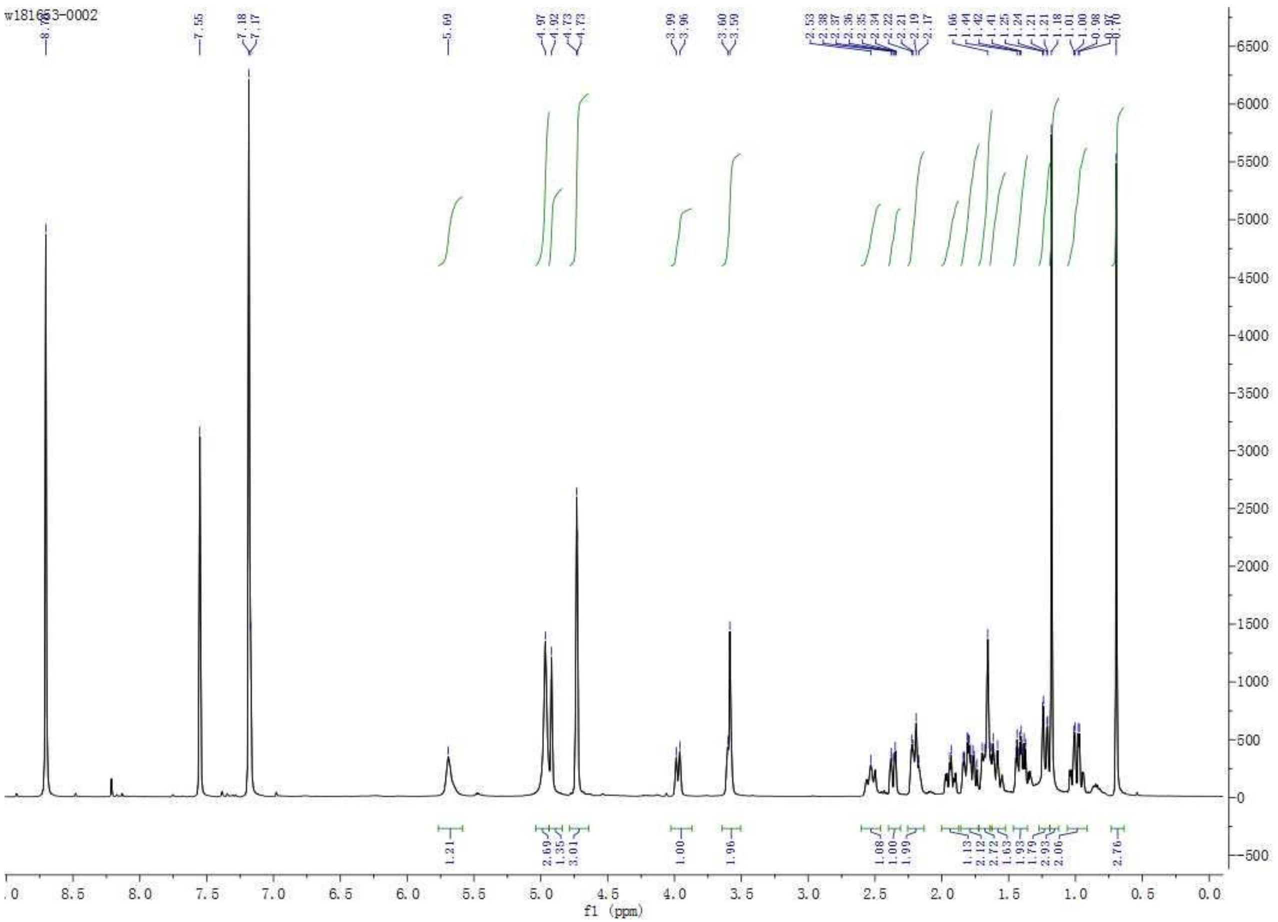

Figure S29. The ^{1}H NMR Spectrum of Compound **10** in pyridine-d_5 (400 MHz)

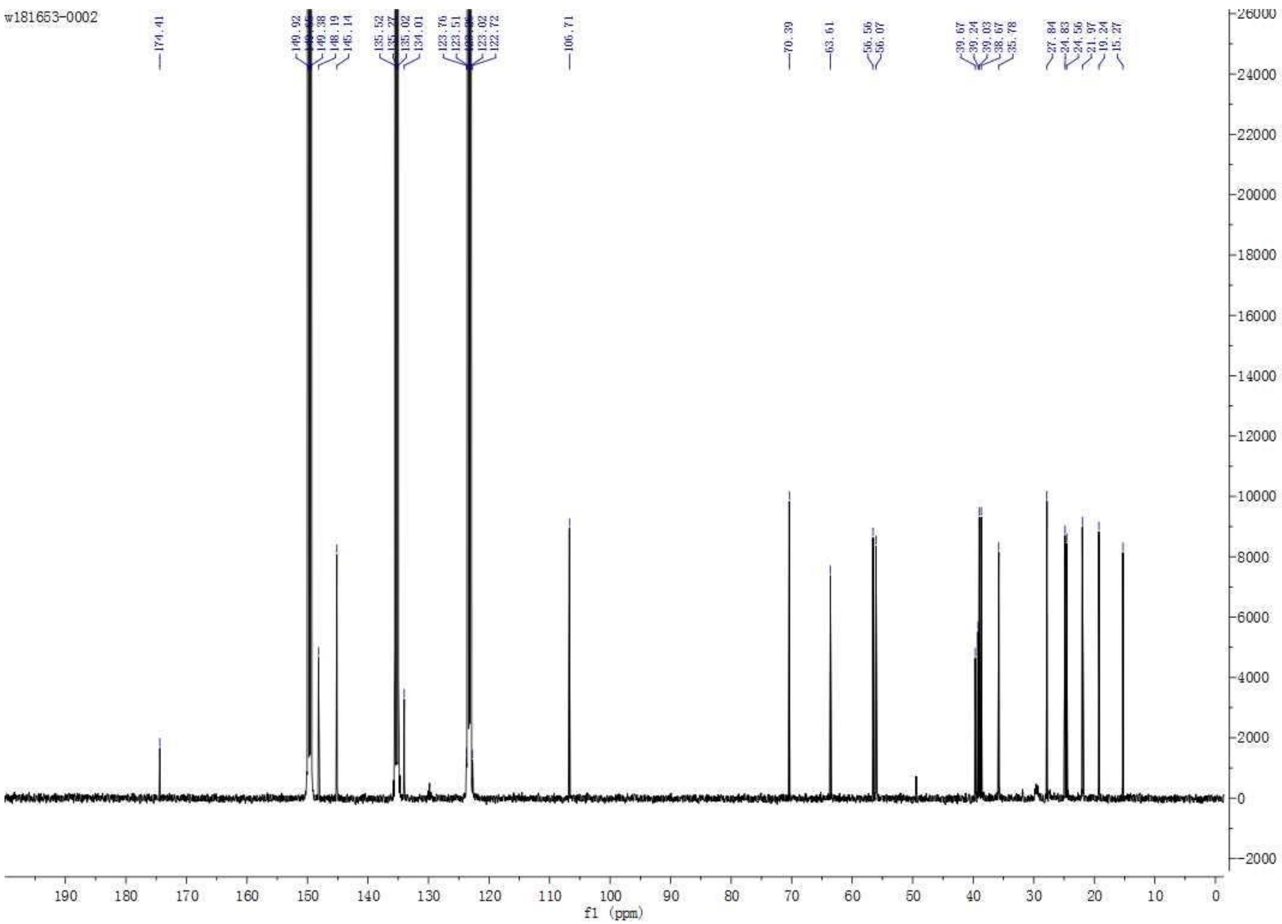

Figure S30. The ^{13}C NMR Spectrum of Compound **10** in pyridine-d_5 (100 MHz)
